# Filling a Gap in Materials Mechanics: Nanoindentation at High Constant Strain Rates up to 10^5^ s^−1^

**DOI:** 10.1002/smll.73215

**Published:** 2026-04-01

**Authors:** Lalith Kumar Bhaskar, Dipali Sonawane, Hendrik Holz, Jeongin Paeng, Peter Schweizer, Jing Rao, Bárbara Bellón, Damian Frey, Aloshious Lambai, Laszlo Pethö, Johann Michler, Jakob Schwiedrzik, Gaurav Mohanty, Gerhard Dehm, Rajaprakash Ramachandramoorthy

**Affiliations:** ^1^ Max‐Planck‐Institute for Sustainable Materials Department of Structure and Micro‐∖Nano‐ Mechanics of Materials Düsseldorf Germany; ^2^ Alemnis AG Thun Switzerland; ^3^ Materials Science and Environmental Engineering Faculty of Engineering and Natural Sciences Tampere University Tampere Finland; ^4^ Current address: VTT Technical Research Centre of Finland Ltd. Espoo Finland; ^5^ Laboratory of Mechanics of Materials and Nanostructures Empa − Swiss Federal Laboratories for Materials Science and Technology Thun Switzerland; ^6^ EPFL, École polytechnique fédérale de Lausanne Institute of Materials (IMX) Lausanne Switzerland; ^7^ Laboratory for High Performance Ceramics Empa − Swiss Federal Laboratories for Materials Science and Technology Dübendorf Switzerland

**Keywords:** fused silica, hardness upturn, high strain rate, instrumentation, molybdenum, nanocrystalline nickel, nanoindentation

## Abstract

A central focus in high strain rate research is understanding the dynamic behavior of materials at strain rates where a strength upturn is observed. While strength upturns at strain rates of 10^3^ to 10^4^ s^−1^ have been widely reported in the literature, their occurrence in certain materials remains controversial, and the underlying physics driving this phenomenon is not yet fully understood. Current mechanical testing methods are limited, as no single technique spans the full strain rate range of 10^1^ to 10^5^ s^−1^ where this phenomenon is expected, and a unified technique would enable consistent post‐deformation characterization with minimal error. To address this, we developed a customized piezoelectric in situ nanomechanical test setup, enabling constant indentation strain rates up to 10^5^ s^−1^, for the first time. Using this system, we examined rate‐dependent hardness in single‐crystalline molybdenum, nanocrystalline nickel, and amorphous fused silica over strain rates from 10^1^ to 10^5^ s^−1^, remarkably revealing a hardness upturn in all three materials. Further, post‐deformation analysis of single‐crystalline molybdenum revealed that the hardness upturn was primarily driven by increased dislocation density, with phonon drag—traditionally considered a dominant contributor playing a minimal role.

## Graphical abstract

A piezoelectric nanoindentation platform enables constant strain‐rate testing up to 10^5^ s^−1^, extending experimental capabilities to extreme deformation regimes. Distinct hardness upturns are revealed across crystalline and amorphous materials, verified as intrinsic responses through quasi‐static reloading. Dislocation analysis in single‐crystalline molybdenum identifies dislocation nucleation, rather than phonon drag, as the governing mechanism at ultrahigh strain rates.

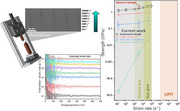

## 1 Introduction

Human civilization is currently in the midst of the Fourth Industrial Revolution, marked by significant advancements in technologies such as high‐speed machining, space vehicles (micro‐asteroid impacts), cold spray, and micro/nanoelectronics (MEMS/NEMS) where materials are exposed to extreme demands, including high strain rates [1, 2]. Despite these technological advancements, characterizing material behavior at high strain rates remains a challenge, as many of the current testing methodologies, especially at the small scales, are still based on quasi‐static approaches. A research gap in the nano‐to‐meso scales persists in reliable quantitative mechanical testing methodologies, particularly for strain rates between 10^2^ and 10^5^ s^−1^. In the macroscale, efforts to study dynamic material behavior have led to the development of techniques like Kolsky (Split‐Hopkinson) bars, gas‐gun driven projectiles, shock impacts, and others [3, 4, 5]. These macroscopic methods, owing to the large sample size, require extreme impact velocities (up to even a few km/s) to generate high strain rates, typically providing reliable data up to 10^4^ s^−1^. Beyond this strain rate, shock effects complicate material response interpretation [6]. In terms of microscale testing, recent micro‐ballistic advancements such as the laser‐induced particle (∼2–40 µm in diameter) impact test (LIPIT) allow material studies at even higher strain rates (10^6^ to 10^9^ s^−1^) [7, 8, 9, 10, 11], providing insights into dynamic hardness and corresponding deformation mechanisms at such extreme strain rates [8, 9, 12]. However, it must be pointed out that quantitative load‐displacement curves cannot be obtained by this technique. In addition, recent efforts have led to the development of miniature Kolsky bar systems to test micro‐scaled pillars [13, 14], enabling testing at higher strain rates in the range of ∼4 × 10^3^ to 10^6^ s^−1^. However, these systems operate just above the critical strain rate regime where the strength upturn is commonly observed, leaving a gap in detailed mechanistic understanding of how materials respond in this transitional range of strain rates.

In terms of material physics, macroscale high strain rate experiments have revealed that many materials show increased strain rate sensitivity, or the strength upturn phenomenon [15, 16, 17, 18], at strain rates between 10^3^ and 10^4^ s^−1^. In metals, this is attributed to a shift from thermally activated dislocation motion at lower strain rates to rate‐dependent dislocation structure evolution and/or dislocation‐phonon drag interactions at higher rates [19, 20, 21]. While the upturn phenomenon is widely accepted, there are still ongoing debates about the exact strain rate at which it occurs and the deformation mechanism responsible. Some investigations from macroscale experiments report the upturn between 10^3^ and 10^4^ s^−1^ strain rate [16, 22, 23] while others suggest that data scatter and experimental artifacts (such as elastic wave propagation) at these rates are responsible for the observed effect [13, 24]. A comprehensive review of this controversy in macroscale high strain rate experiments can be found in the work of Z. Rosenberg et al. [25]. Conversely, the microscale LIPIT technique enables high strain rate explorations only higher than 10^6^ s^−1^, creating a gap in strain rate dependent testing of materials. Both dynamic macroscale and LIPIT microscale techniques allow the high strain rate investigation of materials, but cannot typically maintain constant strain rates during the entire duration of the experiment, further complicating post‐deformation analysis to understand the underlying deformation mechanisms.

Instrumented nanoindentation offers a promising solution for high strain rate testing, enabling constant strain rate experiments at the micron and sub‐micron scales in a variety of material systems while uniquely allowing testing of small phases or features inaccessible to other methods [26, 27]. While traditionally limited to quasi‐static strain rates below 10^−1^ s^−1^, recent efforts have extended nanoindentation to higher rates. Impact‐based techniques, using pendulum actuators [28, 29, 30], can achieve an average high strain rate of 10^4^ s^−1^ but lack precise control over load, displacement, and strain rate, complicating deformation analysis. Advanced nanomechanical systems now allow hardness measurements across strain rates of 10^1^ to ∼10^4^ s^−1^, but these methods face challenges, including the inability to maintain a constant strain rate beyond 10^2^ s^−1^ and difficulties in determining initial step loads for achieving these strain rates, often requiring complex numerical solutions [31, 32, 33, 34, 35]. Displacement‐controlled systems [36, 37, 38] have achieved constant indentation strain rates up to 10^3^ s^−1^, but this remains an order of magnitude below the rates where an upturn in material strength is expected, underscoring the need for further advancements in high constant strain rate nanoindentation techniques.

In this study, we present a piezoelectric‐based in situ high‐speed nanomechanical testing setup capable of achieving constant indentation strain rates ($\dot{h}/h$), for the first time, from 10^1^ s^−1^ up to 10^5^ s^−1^. Such extreme testing also necessitated the development of highly custom‐modified support electronics and experimental protocols for capturing precise load‐displacement data during these high‐strain‐rate indentations. Further, new methodologies were also devised for extracting accurate hardness values from the load‐displacement curves captured at high strain rates. This enabled the assessment of hardness and deformation behavior in single‐crystalline molybdenum, nanocrystalline nickel, and amorphous fused silica at unprecedented constant indentation strain rates. Comprehensive post‐indentation analyses were performed using confocal laser microscopy, quasi‐static reloading, and micro‐pillar compression for fused silica to further validate the observed hardness trends. Moreover, transmission electron microscopy (TEM) of single‐crystalline molybdenum, in conjunction with theoretical calculations, provided new insights into previously unresolved questions in fundamental material science governing deformation behavior at high strain rates. By enhancing our understanding of deformation mechanisms at extreme strain rates, these findings open new avenues for tailoring the mechanical properties of materials through strain‐rate‐dependent microstructural engineering.

## 2 Results and Discussion

### 2.1 Measurement of High Constant Strain Rate Nanoindentation Curves and Upturn in Hardness

**FIGURE 1:**
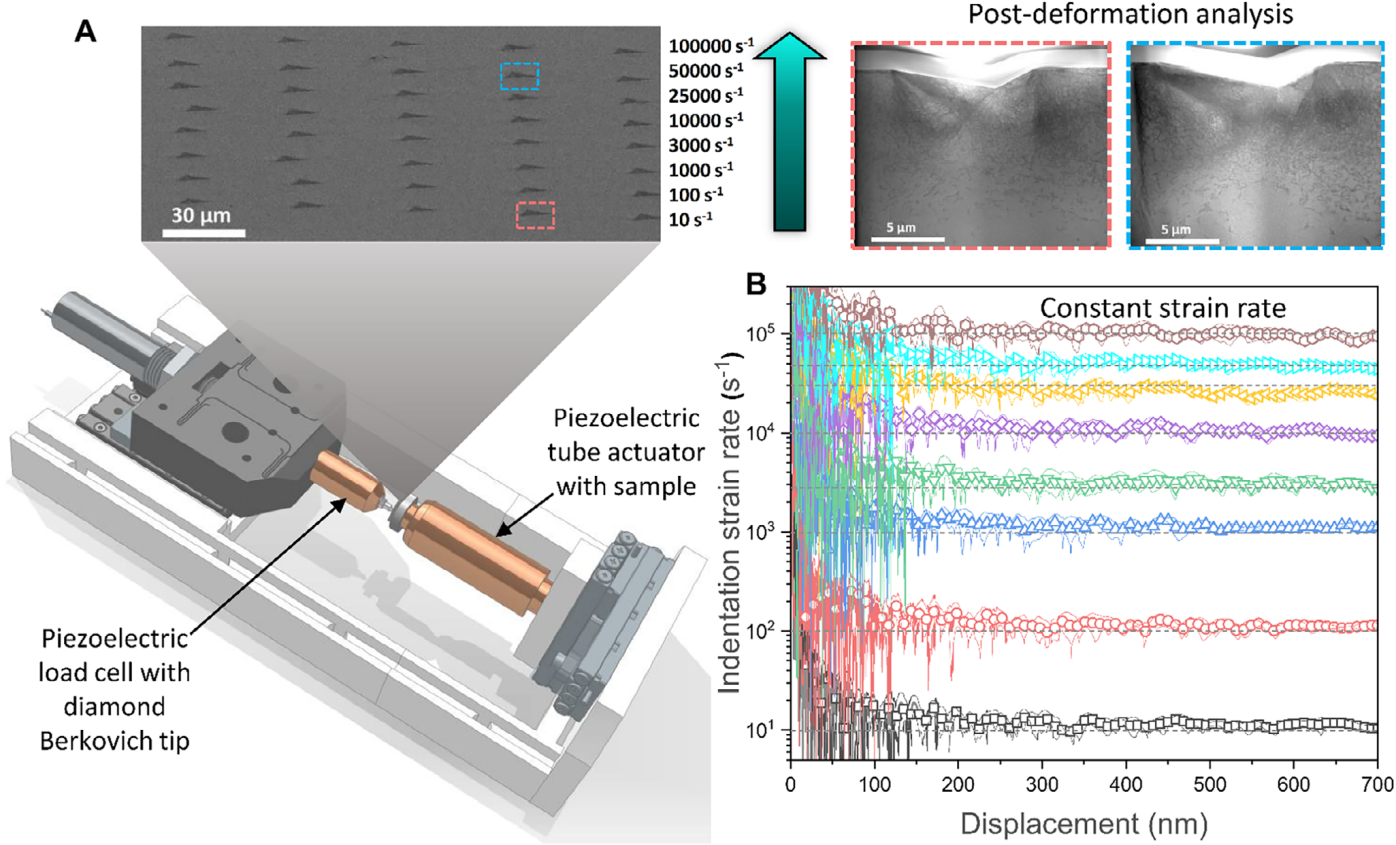
High constant strain rate piezoelectric nanoindentation system: (A) The schematic of the nanomechanical instrument with which nanoindentation tests were conducted over five orders of strain rate, from 10^1^ to 10^5^ s^−1^ and the representative indents on molybdenum across all strain rates. The ability to perform post‐deformation microstructural analysis of the material to investigate the underlying mechanisms at each strain rate individually is additionally shown. (B) The constant indentation strain rate ($\dot{h}/h$) from 10^1^–10^5^ s^−1^ up to an indentation depth of 700 nm.

This study presents, for the first time, high constant strain rate nanoindentation ($\dot{h}/h$) from 10^1^ s^−1^ to 10^5^ s^−1^ using a modified in situ nanomechanical testing platform (Alemnis AG) as shown in Figure 1A. Detailed instrumentation modifications and methods for capturing reliable load and displacement data over short time spans (∼150 µs) are outlined in the Methods section. A significant breakthrough in this work is the ability to maintain a constant indentation strain rate, which enabled accurate investigation of the underlying deformation mechanisms and their correlation with specific strain rates (up to 5 × 10^4^ s^−1^). While the stress state beneath the indenter tip is inherently non‐homogeneous, post‐deformation microstructural analysis was carefully conducted in a consistent spatial region beneath the indent across all strain rates. This ensured that variations in the observed microstructure could be reliably attributed to differences in strain rate, rather than variations in stress localization or sampling position. Although higher indentation depths were tested, the strain rate plot in Figure 1B is limited to 700 nm, beyond which the strain rate drops at the highest tested strain rate of 10^5^ s^−1^, as shown in Supplementary Figure S1. This drop in strain rate beyond 700 nm indentation depth is due to the inability of the high‐speed, high‐voltage amplifier to supply an amplified voltage output with extreme voltage gradients (slew rate > 75 V/µs) needed to maintain a constant strain rate of 10^5^ s^−1^. To maintain consistency, all further results and analyses presented here are limited to 700 nm depth across all strain rates.

**FIGURE 2:**
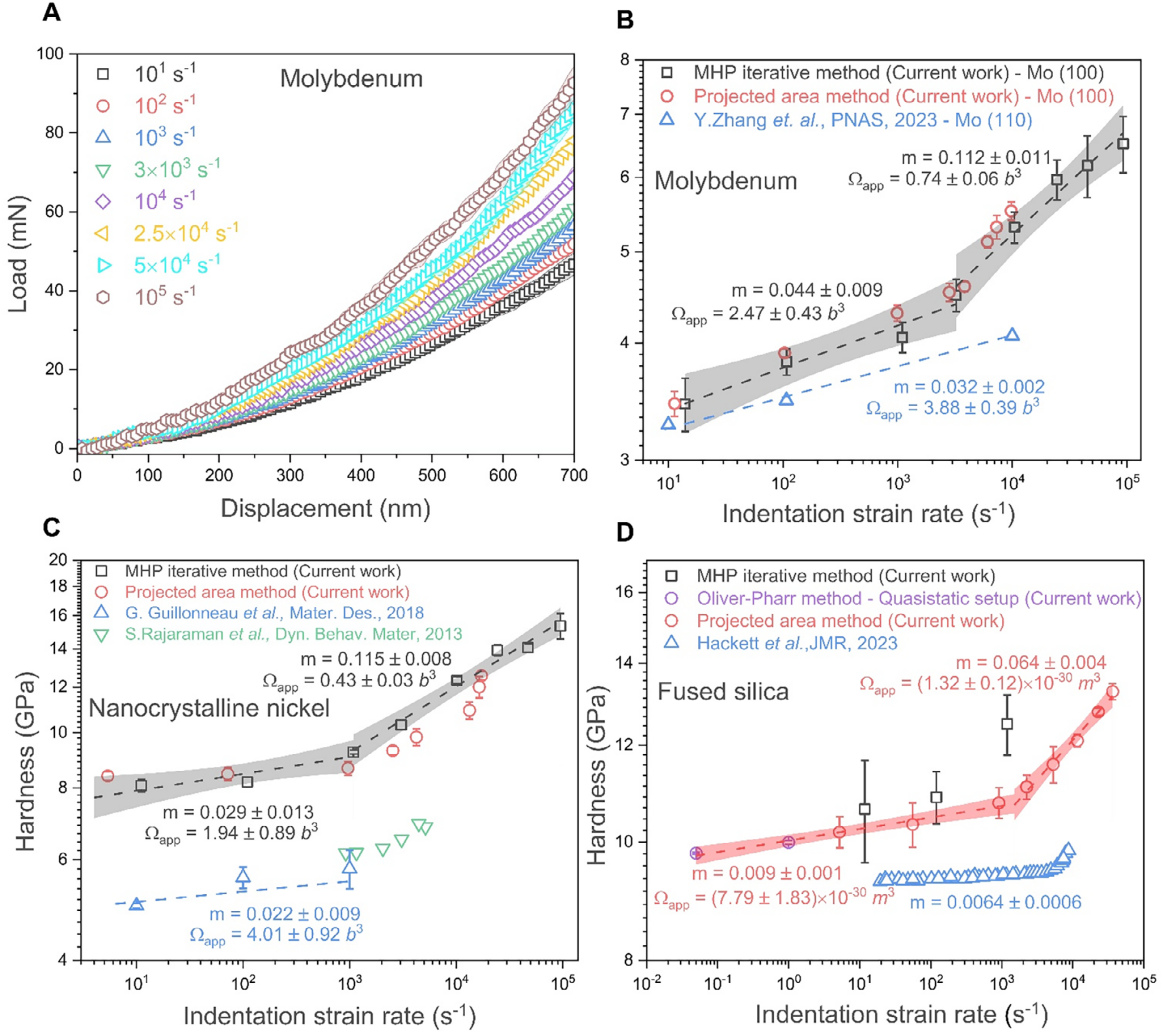
Hardness upturn in single crystalline molybdenum, nanocrystalline nickel, and amorphous fused silica: (A) Load‐displacement curve for single‐crystalline molybdenum in the loading segment after applying all corrections, and hardness values for (B) molybdenum, (C) nanocrystalline nickel, and (D) fused silica, obtained using both the iterative and projected area methods and compared with literature values. Both the axes in (B–D) are in log scale. The strain rate sensitivity (*m*) was calculated using the formula, $m=\frac{\partial lnH}{\partial ln\dot{\varepsilon }}$, where *H* is hardness and $\dot{\varepsilon }$ is strain rate. Similarly, the apparent activation volume (Ω_app_) was calculated using the formula, $\Omega_{app}=\sqrt{3}Ck_{B}T\frac{\partial ln\dot{\varepsilon }}{\partial H}$, where *C* is the proportionality constant and equal to 3, *k_B_* ​ is the Boltzmann constant, T is temperature, H is hardness and $\dot{\varepsilon }$ is strain rate. The results in figures (B & C) are expressed in terms of the Burgers vector *b*, which is 0.272 nm for molybdenum and 0.249 nm for nanocrystalline nickel, whereas for fused silica in (C) is expressed in cubic meters. The bands are with 95% confidence index. All the data shown represent the mean of five independent measurements, with the corresponding standard deviation indicated.

The corrected load‐displacement curves for single crystalline molybdenum obtained after applying all protocols outlined in the Methods Section are shown in Figure 2A, while those for nanocrystalline nickel and amorphous fused silica are presented in Supplementary Figure S2. In the load‐displacement curves, only the loading portions are shown, as resonance effects from various components of the testing system significantly affect the unloading segments, as shown in Supplementary Figure S3. Consequently, the traditional Oliver‐Pharr method [26] for hardness determination was inapplicable. Instead, two other complementary techniques, the Merle‐Higgins‐Pharr (MHP iterative method) [39] and projected area methods, were used (Supplementary Information ‐ Section S3.4). Figure 2B–D presents rate‐dependent hardness values for molybdenum, nanocrystalline nickel, and fused silica obtained from both methods, compared against literature values. Supplementary Figure S4A–C shows hardness vs displacement for all three materials using the MHP iterative method.

Using the MHP iterative method, hardness values were determined across the full displacement range; thereafter, the Nix‐Gao model [40] was applied to fit data between 200–700 nm for molybdenum and nanocrystalline nickel, where strain rates remained constant for all strain rates, before being extrapolated for direct comparison with hardness values obtained using the projected area method. Extrapolation to larger depths follows standard practice, where the Nix–Gao framework is applied within the strain‐gradient‐dominated regime and extended to estimate the asymptotic hardness at greater depths [41]. In contrast, the projected area method provides a single hardness data point only at the highest load. Supplementary Figure S5A indicates that at the highest load, the strain rate is lower than the applied strain rate. Consequently, the hardness values from the projected area method in Figure 2B–D were adjusted to correspond to the strain rate ($\dot{P}/2P$ – from load), calculated by averaging over a range of 10–20 nm at the peak load. In this work, for hardness calculations using the projected area method, the peak load after all corrections (refer to the method Section S4.1.1 – Methodology for signal acquisition and synchronization) was used (as shown in Supplementary Figure S5B). Although nanocrystalline nickel is generally not expected to exhibit an indentation size effect [42], the Nix–Gao model was nonetheless employed for this material as well. The rationale for this choice is that the hardness did not saturate at depths below 700 nm—a behavior similar to what has been reported in prior studies [43]. Only beyond 700 nm did hardness show saturation. Supporting evidence can be seen in Supplementary Figure S4D,E, which display hardness data for both nanocrystalline nickel and molybdenum up to 1000 nm. The enlarged view hardness data between 700 and 1000 nm for nanocrystalline nickel and molybdenum is shown in Supplementary Figure S4F,G, respectively. A guide‐for‐the‐eye horizontal dotted line helps visualize that hardness for nanocrystalline nickel stabilizes beyond 700 nm, while molybdenum does not, due to indentation size effect. Additionally, Supplementary Figure S4H presents hardness values for nanocrystalline nickel obtained both by averaging over depths of 600–650 nm and by applying the Nix–Gao model (200–700 nm range) with extrapolation. While the relative trends match, the absolute values from the averaging method are higher. Using greater depths for averaging is not feasible at the highest strain rate (10^5^ s^−1^), as strain rate drops at depths greater than 700 nm (Figure S1), which, as a result, is reflected in hardness (Figure S4D,E).

However, for fused silica, no Nix–Gao correction was applied since it does not exhibit indentation size effects and the assumptions underlying the Nix–Gao model [40] (e.g., dislocation‐based hardening) are not valid for amorphous glassy systems [41]. Instead, hardness values were averaged over a depth range of 600–650 nm. Additionally, Figure 2D presents hardness measurements for fused silica at strain rates of 5 × 10^−2^ s^−1^ and 10^0^ s^−1^, obtained using two independent systems – KLA G200 and the quasi‐static system from Alemnis AG [37], respectively (The details of the experiments are given in Supplementary Information Section S3.1 (Nanoindentation experiments)). The observed hardness trends are consistent and align well with those obtained using the modified high strain rate indentation setup, further validating both the modified high strain setup and the methodology used for hardness measurement.

In Figure 2B, the hardness trends for molybdenum exhibit a strong correlation between the two complementary methods, with a distinct upturn in hardness observed at strain rates exceeding 3 × 10^3^ s^−1^. Beyond this strain rate, the calculated strain rate sensitivity (given by, $m=\frac{\partial lnH}{\partial ln\dot{\varepsilon }}$, where *H* is hardness and $\dot{\varepsilon }$ is strain rate) increases approximately− 2.5 times from m = 0.044 ± 0.009 to 0.112 ± 0.011. Previous high strain rate impact nanoindentation study up to 10^4^ s−^1^ on molybdenum [31] with (110) orientation showed a similar value of strain rate sensitivity (m = 0.032 ± 0.002). However, no hardness upturn was reported in this study for the (100) orientation. This variation could be attributed to differences in the sample orientation and the testing methodology used in the literature, where experiments resulted in varying strain rates and instantaneous strain rates were used to report hardness values [31].

Similarly, for nanocrystalline nickel in Figure 2C, the hardness values from both techniques showed good agreement, with an upturn observed at strain rates above 10^3^ s^−1^ and an approximately four‐fold increase in strain rate sensitivity (from *m* = 0.029 ± 0.013 to 0.115 ± 0.008). Literature reports of constant strain rate indentation up to 10^3^ s^−^^1^ showed similar rate sensitivity (*m* = 0.022 ± 0.009) [37]. Notably, the upturn in hardness observed in this work aligns with dynamic compression experiments using Kolsky bars [44], which also reported a strength increase above 10^3^ s^−1^. While the grain sizes in this study (38 nm) are comparable to those reported in the literature (17 nm [44] and 26 nm [37]), the variations in absolute hardness values in Figure 2C are likely due to chemical impurities introduced by electrodeposition conditions, which are known to affect the microstructure and, consequently, the mechanical properties [45]. For fused silica, unlike molybdenum and nanocrystalline nickel, the hardness and strain rate sensitivity values from the two complementary techniques showed differences from 10^3^ s^−1^ and above as shown in Figure 2D. Interestingly, the iterative method did not provide any solutions for hardness above 10^3^ s^−1^. The strain rate sensitivity (*m*) was calculated as 0.009 ± 0.001 up to strain rates of 3 × 10^3^ s^−1^, consistent with literature [46]. Beyond this threshold, however, an upturn in hardness was observed, with a strain rate sensitivity of 0.064 ± 0.004 — a 7‐fold increase.

### 2.2 Analysis of the Deformation Mechanism Behind the Hardness Upturn

The literature data [34] for fused silica also shows a similar upturn in hardness. However, authors in that study expressed caution, suggesting it might be a data processing artifact caused by filtering rather than a genuine material response. There are two key questions to be answered for fused silica: (i) why did the iterative method fail to provide a hardness solution beyond 10^3^ s^−1^, and (ii) is the observed upturn in hardness a true material behavior?

**FIGURE 3:**
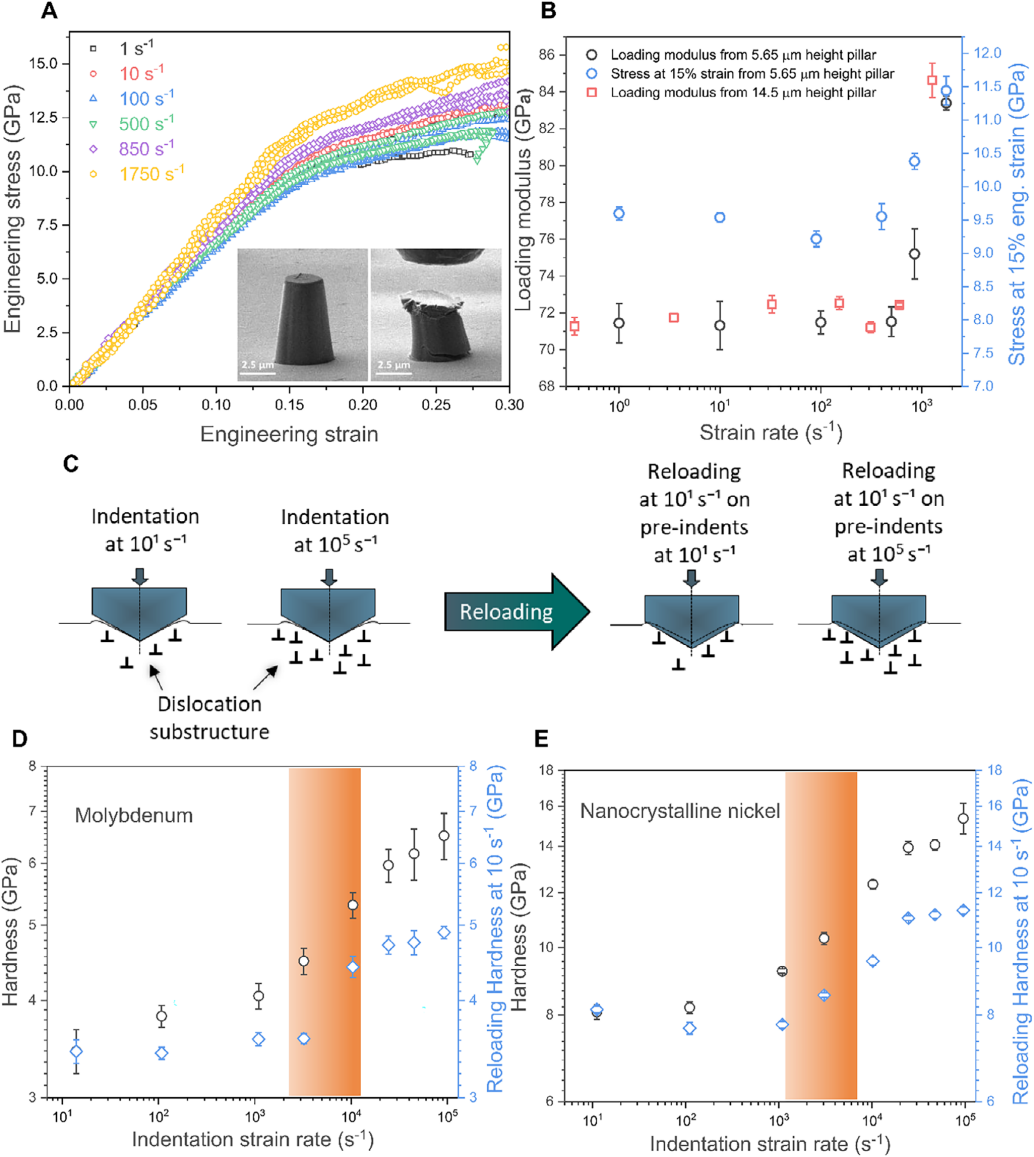
Validation of hardness upturn: (A) Shows the engineering stress–strain curves for fused silica micropillars of 5.65 µm height tested at strain rates ranging from 1 to 1750 s^−1^. (B) shows quantitative analysis of loading modulus (for 5.65 and 14.5 µm height fused silica pillar) and flow stress at 15% engineering strain (for 5.65 µm height fused silica pillar), showing significant changes at strain rates of ∼10^3^ s^−1^ and beyond. (C) shows the schematic of the reloading process, where all reloads on pre‐indents at various strain rates were conducted using a profile of 10^1^ s^−1^. (D & E) shows reloading hardness trends for molybdenum and nanocrystalline nickel, respectively, with hardness vs strain rate trends overlaid for clarity. The strain rate at which the hardness upturn is observed is marked by an orange band and the hardness values are in log scale. All the data shown represent the mean of five independent measurements, with the corresponding standard deviation indicated.

Fused silica, characterized by its high free volume due to low atomic packing density, predominantly deforms through densification [47]. Upon sufficient densification, it also exhibits conservative shear flow as a plastic deformation mode [48]. Literature reports indicate a maximum densification of 21%, with changes in elastic modulus becoming noticeable once densification exceeds 5% [48]. Interestingly, in this study, from micropillar compression experiments pristine fused silica pillars exhibited an increase in apparent loading modulus from 71.4 ± 0.9 to 83.4 ± 0.4 GPa under compression at strain rates exceeding ∼10^3^ s^−1^ as shown in Figure 3A,B. This is hypothesized to occur because, at high strain rates, the intense hydrostatic forces generated by the taper of the fused silica micropillars may induce reversible instantaneous densification, potentially leading to a change in elastic modulus. To clarify, densification certainly occurs in the plastic regime; however, in this study the observed modulus change appears within the elastic region, and the hypothesis is therefore framed on that basis. Although this hypothesis aligns with literature evidence that supports an increase in elastic modulus with densification [48, 49], the direct experimental evidence of densification remains elusive, as it occurs within the elastic region. However, similar trends in loading modulus have been reported in other compression studies on fused silica at comparable strain rates [36]. It should be noted that the measured modulus is referred to as the loading modulus rather than the true elastic modulus, since taper from fabrication, frictional effects, misalignment, and other experimental artifacts could potentially influence the results.

Further to rule out the possibility of a machine‐induced artifact causing the observed modulus change, compression tests were conducted on fused silica pillars of two different heights−5.65 and 14.5 µm. Notably, both pillar geometries showed a change in loading modulus at the same transition strain rate of ∼10^3^ s^−1^ (Figure 3B). At this strain rate, the corresponding compression velocities were 5.65 mm/s for the 5.65 µm pillars and 14.5 mm/s for the 14.5 µm pillars. Furthermore, the hardness upturn observed in indentation experiments (Figure 2D) aligns with the flow stress upturn seen in the micropillar compression results at similar strain rates (Figure 3B). Specifically, this transition corresponds to an instantaneous indentation velocity of just 0.7 mm/s for a 700 nm indentation depth at a strain rate of 10^3^ s^−1^, reinforcing that the observed upturns are consistent across different testing methods on the same material, thereby further ruling out machine‐related artifacts. The stress‐strain curves for the 5.65 µm high pillars are given in Figure 3A, and those of the 14.5 µm high pillars are shown in the Supplementary Figure S6.

The observed modulus changes in fused silica micropillars beyond ∼10^3^ s^−^^1^ can explain why the iterative hardness estimation method fails, as it relies on the assumption of a constant reduced modulus across strain rates (Supplementary Information Section S3.4.2 (Hardness measurement). However, the reason for the observed upturn in nanoindentation hardness, while potentially attributable to modulus changes, remains speculative and could also result from a shift in deformation mechanisms, as the depth profiles of confocal microscopy images of fused silica across strain rates (Supplemenary Figure S7A) reveal small pile‐ups at lower strain rates, indicating a potential shear flow mechanism [48], whereas these pile‐ups are absent at higher strain rates. These trends are consistent with previous findings in glassy materials, where high strain rate‐induced transitions from shear band propagation to shear band nucleation [50], or alternatively, structural responses governed by the time scale of crack propagation in brittle materials [51], have been proposed. Despite these compelling parallels, the underlying mechanism remains unresolved. Significantly, even the loading modulus in this study exhibits clear strain‐rate sensitivity, in addition, other effects such as indenter tip heating at high strain rates or local fracture could also influence, highlighting the need for further investigation into the high strain rate behavior of fused silica using advanced characterization techniques such as synchrotron‐based ptychographic X‐ray computed tomography (PXCT) [52], coupled with atomistic based continuum‐scale simulations.

Unlike fused silica, the line profiles for molybdenum and nanocrystalline nickel showed no significant difference in pile up across strain rates, as illustrated in Supplementary Figure S7B,C. The observed upturn in hardness in these materials should correlate with changes in the microstructural or dislocation substructure beneath the indent. To confirm this, the same indents formed during high strain rate indentation were reloaded [31] at a lower strain rate of 10^1^ s^−1^. Details of this reloading process along with hardness estimation are provided in Supplementary Information Section S3.3 (Quasi‐static reloading experiments), and a simplified schematic of the procedure is shown in Figure 3C. To further clarify the concept of reloading, during the first loading dislocations and deformation substructures are created in an otherwise undeformed material, whereas reloading probes the response of this newly created substructure. The hardness obtained during reloading, along with the hardness trend as a function of strain rate for molybdenum and nanocrystalline nickel, is presented in Figure 3D,E, along with the load‐displacement curves in Figure S8A,B. Notably, the strain rate at which the upturn in hardness occurs coincides with a distinct increase in reloading hardness, indicating a possible change in microstructural/dislocation substructure. Similar trends were observed in the reloading hardness of fused silica, as shown in Figure S8C.

### 2.3 Rate Dependent Microstructure Evolution in Metals

To better understand the rate‐dependent deformation mechanism in metals, a combination of thermal activation analysis, microstructural characterization via TEM, and theoretical calculations was conducted. The apparent activation volume (*Ω_app_*) was calculated and expressed in terms of the Burgers vector (*b*), as shown in Figure 2B,C. The Burgers vector (*b*) is 0.272 nm for molybdenum and 0.249 nm for nanocrystalline nickel [53]. Before the hardness upturn, for molybdenum, *Ω_app_* was 2.47 ± 0.43 *b* ^3^, consistent with literature values at room temperature [54], indicating the formation and migration of dislocation kink pairs typical for BCC metals. For nanocrystalline nickel, *Ω_app_* was 1.94 ± 0.89 *b* ^3^, reflecting grain‐boundary mediated deformation typical for FCC nanomaterials, aligning well with reported values [42, 55]. However, beyond the hardness upturn, the apparent activation volumes decreased significantly, with molybdenum and nanocrystalline nickel showing 0.74 ± 0.06 *b* ^3^ and 0.43 ± 0.03 *b* ^3^ respectively. Interestingly, such low activation volumes suggest the rate dependence is due to dislocation nucleation events [56, 57]. In Figure 2D, the activation volume for fused silica is expressed in cubic meters rather than in terms of the Burgers vector (*b*). This is because the concept of activation volume originates from dislocation‐mediated plasticity, where it represents the volume swept by collective dislocation motion during deformation. Since fused silica deforms primarily through densification and shear flow rather than dislocation activity, the assumptions underlying activation volume analysis are not fully applicable, and thus the value is reported in cubic meters.

Two main theories have been proposed in the literature to explain the physical origin of the hardness upturn at high strain rates. The first attributes the effect to the transition in deformation mechanisms: At low strain rates, thermally activated dislocation glide governs plastic flow, dislocations overcome atomic obstacles with the aid of thermal vibrations. As the strain rate increases, the time available for thermal activation decreases, requiring higher applied stresses for dislocation glide and thus raising hardness. According to the Orowan relation, the plastic strain rate is limited by the available dislocation density (*ρ*), and the maximum dislocation velocity (*ν_max_*). Once the imposed strain rate approaches this upper bound (${\dot{\varepsilon }}_{max}^{p}=\rho b\nu_{max}$), the existing dislocations are insufficient to accommodate plastic flow. It was observed from Discrete dislocation dynamics (DDD) simulations that if the applied strain rate surpasses this threshold, the existing dislocations beneath the indent become insufficient to accommodate the imposed stress, resulting in a substantial increase in dislocation nucleation— a phenomenon referred to as the exhaustion zone [19]. Similar theories were proposed by Follansbee et al. [16], based on macroscale high‐strain‐rate experiments. However, obtaining direct evidence of an increase in dislocation density remained challenging, as only an approximately 1.5 fold increase was expected [16] from theoretical equations, and earlier attempts using transmission electron microscopy were not successful in detecting significant changes in dislocation densities accurately [58].

The second theory emphasizes the role of dislocation–phonon interactions. At sufficiently high strain rates, phonon drag imposes a velocity‐dependent resistance to dislocation motion, contributing to rate‐dependent strengthening. However, constitutive modeling by Tang et al. suggests that dislocation‐phonon drag, a prominent mechanism considered responsible for the upturn in strength, becomes significant only at strain rates exceeding 10^5^ s^−1^, whereas the upturn observed at 10^3^ s^−1^ is primarily attributed to thermal activation effects [15].

**FIGURE 4:**
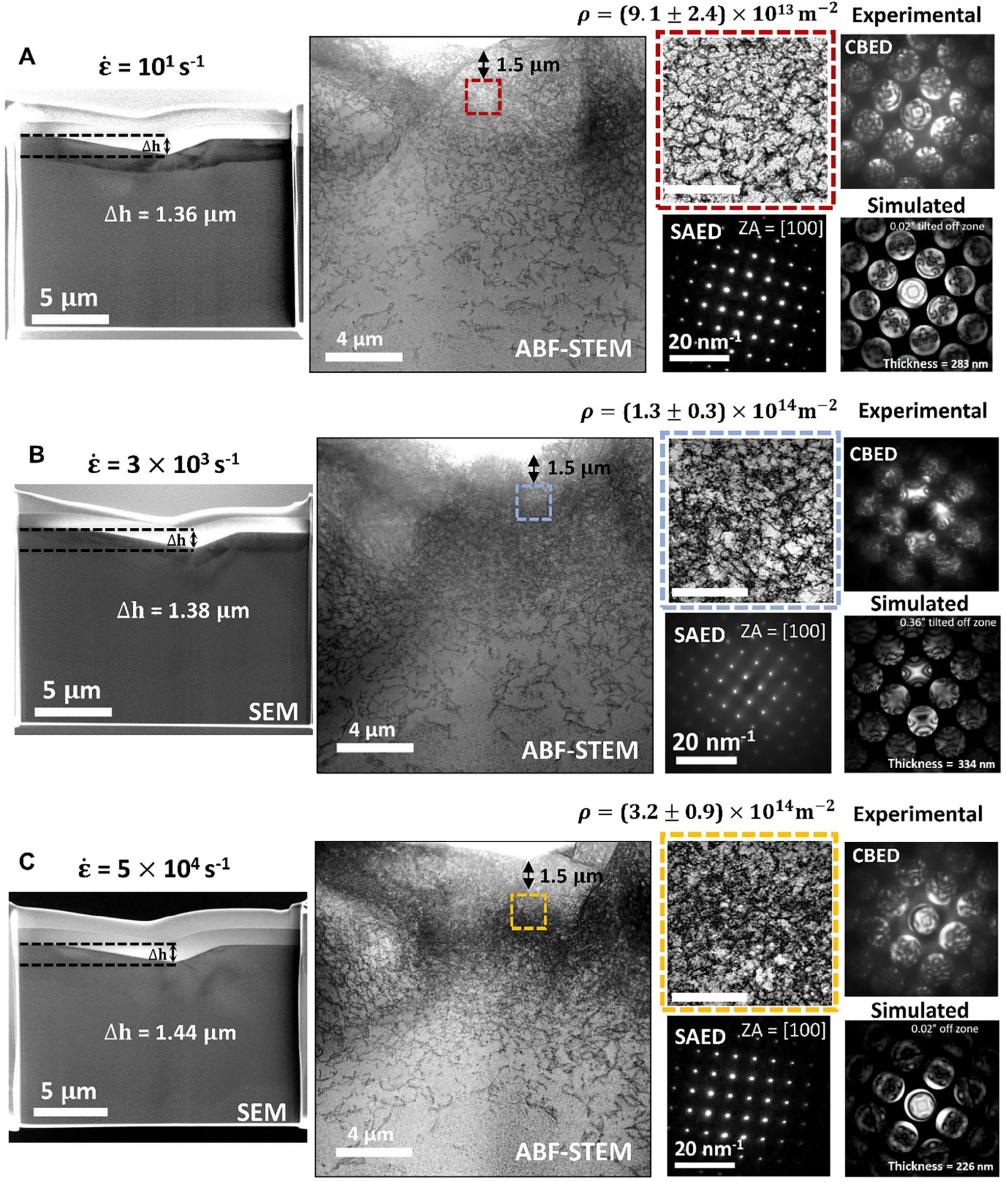
Reason for hardness upturn in single crystalline molybdenum: Scanning electron microscopy (SEM) images, annular bright field scanning transmission electron microscopy (ABF‐STEM) images, selected area electron diffraction (SAED), and experimental and simulated convergent beam electron diffraction (CBED) images for molybdenum indents at strain rates of (A) 10^1^ s^−1^, (B) 3 × 10^3^ s^−1^, and (C) 5 × 10^4^ s^−1^. The SEM images display indentation depths across strain rates, while the ABF‐STEM images highlight the region (approximately 1.5 µm away from the indent tip) used for dislocation density measurements. The scale bar in highlighted regions correspond to 750 nm. The dislocation density values and TEM lamella thickness calculated from the ABF‐STEM images and simulated CBED.

To investigate these claims, a detailed post‐deformation microstructural analysis of indents in molybdenum was performed using TEM. Molybdenum was selected due to its single‐crystalline structure, which facilitates a simpler interpretation compared to nanocrystalline nickel. To identify the underlying deformation mechanisms, TEM analysis was done on the indents conducted at the lowest tested strain rate ‐ 10^1^ s^−1^, just before the upturn at 3  ×  10^3^ s^−1^, and at the highest strain rate 5  ×  10^4^ s^−1^ at which the strain rate remains constant for the majority of the indentation depth. Figure 4A–C presents annular bright field scanning transmission electron microscopy (ABF‐STEM) images of indents at three different strain rates, revealing clear qualitative differences in dislocation density. The dislocation densities were also quantified, with details provided in the Supplementary Information Section S3.5 (TEM and dislocation density calculations).

The measured values are (9.1 ± 2.4) ×  10^13^ m^−2^, (1.3 ± 0.3) × 10^14^ m^−2^, and (3.2 ± 0.9) × 10^14^ m^−2^ at strain rates of 10^1^ s^−1^, 3 × 10^3^ s^−1^, and 5 × 10^4^ s^−1^ respectively, at a distance of 1.5 µm from the tip of the indent, showing an approximately 3‐fold increase in dislocation density across strain rates. Notably, scanning electron microscopy (SEM) images of the indent cross‐sections in Figure 4 A–C indicates that the indentation depth remains nearly constant across all three strain rates. Dislocation densities were notably measured at a consistent distance of approximately 1.5 µm from the indent apex across all strain rates. This depth was chosen for ease of measurement and to confirm the relative trends across different strain rates, and also, dislocation density measurement very close to the indent was difficult. It is to be noted that dislocation densities would be substantially higher near the indenter tip. However, this study represents the first instance where dislocation density at such high strain rates has been imaged and comparatively quantified across strain rates with absolute certainty. A further, detailed dislocation density analysis from 1 to 3 µm indentation depthat 1 µm intervals over 1 × 1 µm^2^ areas is provided in the Supplementary InformationSection S3.5 (TEM and dislocation density calculations, and Supplementary Figure S21).

**FIGURE 5:**
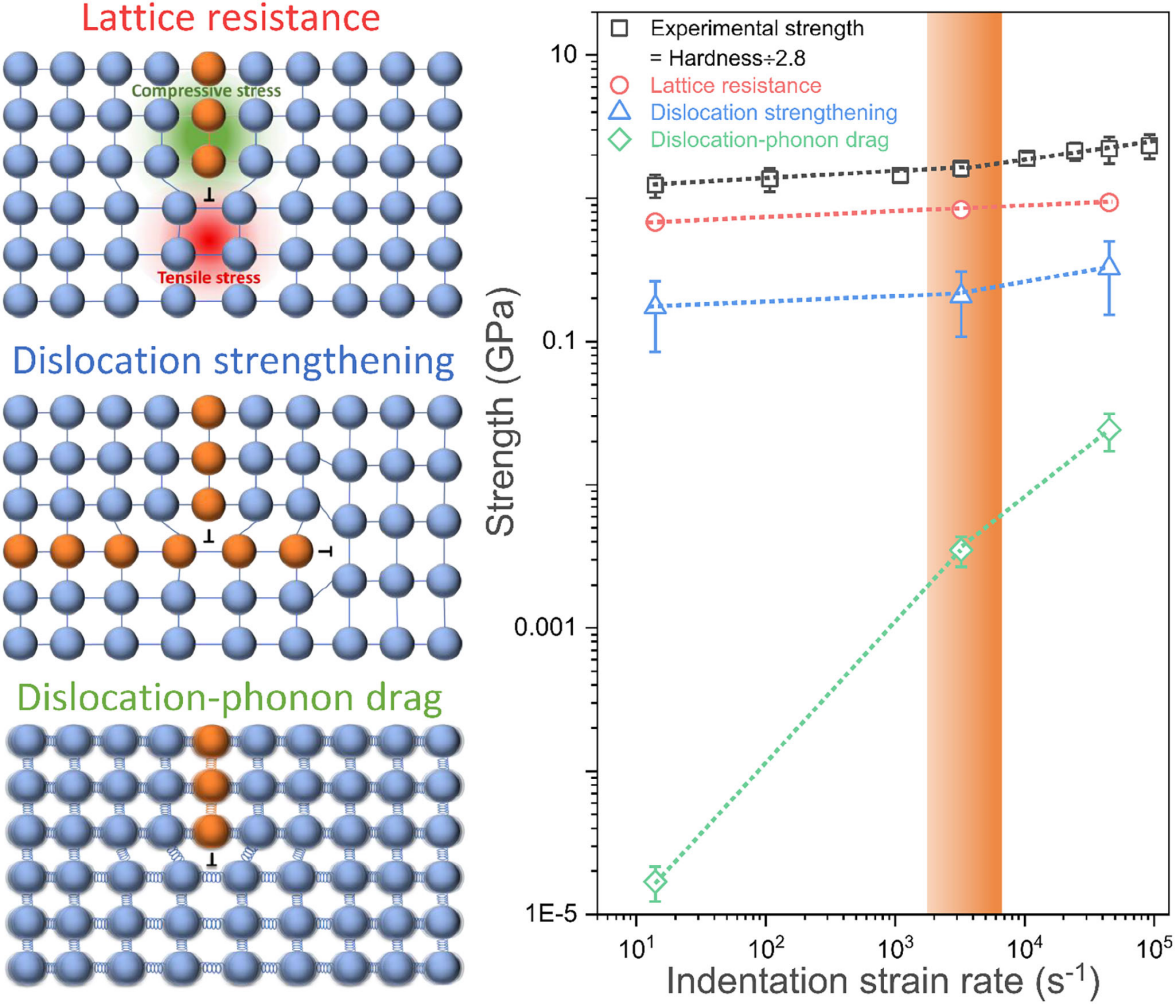
Deformation mechanism behind hardness upturn: The trends of potential strengthening mechanisms for single‐crystalline molybdenum, including lattice resistance, dislocation strengthening, and dislocation‐phonon drag. The measured hardness values, normalized by dividing by a constraint factor of 2.8, are plotted alongside the calculated strengths for comparison. A guide to the eye is provided in all strengthening mechanisms. The strain rate at which the strength upturn is observed is marked by an orange band and the strength values are in log scale. Schematics illustrating each strengthening mechanism are also included.

With the dislocation density values determined, we used them to investigate the possible deformation mechanism responsible for the observed upturn in hardness. Since the material under consideration is single crystalline, the possible strengthening mechanisms are limited to lattice resistance strengthening, dislocation strengthening, and dislocation drag. The individual contributions were evaluated using experimentally measured microstructural parameters and theoretical calculations, enabling quantitative decoupling of competing mechanisms and the identification of the dominant term governing the hardness upturn. Detailed calculations for each of these mechanisms are provided in – Supplementary Information Section S1 (Strengthening mechanism), and the resulting trends are plotted in Figure 5 across these three strain rates. For comparison, the measured hardness values were normalized by dividing by a constraint factor of 2.8 and plotted alongside the calculated strengths. Figure 5 clearly shows that dislocation strengthening is the dominant mechanism driving the upturn in strength. Specifically, at a strain rate of 5 × 10^4^ s^−1^, the strength attributed to dislocation hardening is approximately 1.6 times greater than that at 3  × 10^3^ s^−1^. Additionally, Figure 5 shows that dislocation drag, previously hypothesized in the literature as a significant contributor to the upturn in strength, has a negligible impact. Even at strain rates as high as 5 × 10^4^ s^−1^, its contribution is minimal, with a value of only 0.024 GPa. By explicitly separating and comparing the individual strengthening contributions, the analysis identifies strain‐rate‐dependent dislocation density evolution as the primary mechanism governing the hardness upturn in single‐crystalline molybdenum.

## 3 Conclusions

This study presents the development of a specialized high‐speed piezoelectric‐based micromechanical testing setup for constant strain rate nanoindentation, achieving unprecedented strain rates from 10^1^ s^−1^ to 10^5^ s^−1^. Custom electronics and advanced protocols ensured the acquisition of accurate load‐displacement signals, enabling reliable hardness measurements using two complementary methods. The rate‐dependent hardness trends of single‐crystalline BCC molybdenum, FCC nanocrystalline nickel and amorphous fused silica were investigated. Surprisingly, all materials demonstrated a hardness upturn with increasing strain rate. The hardness upturn was validated through a combination of micropillar compression tests in fused silica and reloading experiments for all materials. The constant strain rate capability allowed a precise correlation of microstructural evolution underneath the indent to the hardness upturn. TEM studies at strain rates exceeding the upturn threshold revealed an increased dislocation density due to dislocation nucleation, offering direct evidence of strain rate‐dependent strengthening mechanisms in single‐crystalline molybdenum with negligible contributions from dislocation‐phonon drag. While the exact rate‐dependent microstructural evolution for nanocrystalline nickel and fused silica still need to be explored, the possibility of conducting high constant strain rate nanoindentation at a wide range of strain rates with reliable load‐displacement curves, hardness data, and rate‐specific microstructural investigations is a new pathway for conducting more accurate and quantitative dynamic material property investigations. Additionally, nanoindentation at such extreme strain rates on individual phases, grains, and grain boundaries, would provide valuable rate‐dependent input for simulations like DDD and enable the development of more accurate physics‐based models in the future [19] for several materials. Furthermore, beyond hardness measurements, the availability of high‐fidelity load–displacement curves at extreme strain rates opens the possibility of coupling high strain‐rate indentation data with FEM‐assisted inverse analysis to reconstruct stress–strain relationships [59, 60] under dynamic conditions, which are otherwise difficult to obtain using conventional macroscale high‐speed mechanical testing due to machine‐related artifacts.

## 4 Materials and Methods

The molybdenum sample, with 99.999% purity and a (100) crystallographic orientation, was commercially sourced from Goodfellow Cambridge Limited. It was progressively polished resulting in a mirror‐like surface finish. The nanocrystalline nickel used in this study was electrodeposited in a UV‐LIGA mold on a silicon wafer using a nickel sulfamate bath. The top surface was polished, and samples were subsequently extracted from LIGA molds. Optical‐grade amorphous fused silica was obtained from MTI Corporation. Furthermore, in this work compression tests were performed on lithography made fused silica micropillars, which had dimensions of approximately 2.5 µm in diameter and 5.65 µm in height, and 5.5 µm in diameter and 14.5 µm in height. The detailed fabrication procedure for fused silica micropillars is described in the work of R. Ramachandramoorthy et al. [36].

### 4.1 Ultrahigh Strain Rate Nanomechanical Testing System

The system used in this study is based on a platform from Alemnis AG, which was modified to further extend the range for high constant strain rate ($\dot{h}/h$) nanoindentation from 10^4^ s^−1^ to 10^5^ s^−1^. A schematic of the modified system is provided in Supplementary Figure S9A,B. The system includes a piezoelectric load cell (Alemnis AG) for load measurements, providing a resolution of approximately 15 µN. The piezoelectric load cell is designed with four electrodes positioned on a concentric cylinder, and the signals from each electrode are collected individually. The signals are then amplified via a high‐impedance charge amplifier (Alemnis AG) with a cut‐off frequency of ∼300 kHz. For actuation, a piezoelectric tube actuator (Alemnis AG) was employed to achieve constant indentation strain rates ($\dot{h}/h$) upto 10^5^ s^−1^. The piezoelectric tube actuator features a low capacitance of 14.5 nF and a high stiffness of 19.14 N/µm. The piezoelectric tube actuator can reach very high velocities of ∼100 mm/s, with a maximum displacement of 4 µm. To attain such high actuator velocities, an external high‐speed high‐voltage amplifier (WMA‐200) from Falco Systems was used. The voltage amplifier can deliver output voltages of ± 175 V, a maximum current output of 150 mA, and has a high slew rate of 80 V/µs (no load), which is crucial for maintaining such high constant strain rates. The voltage amplifier is capable of receiving shaped input profiles with a maximum amplitude of ± 10 V, which are then amplified 20‐fold for output. Additionally, the voltage amplifier is equipped with a monitoring port that continuously records the amplified voltage albeit reduced by 100 times in magnitude, which was critical for the synchronization of different load and displacement signals, as detailed further in this section. To measure the actuator's displacement during indentation testing, two piezo‐resistive strain gauges were attached on opposite sides of the piezoelectric tube, and a full Wheatstone bridge was formed using an external completion circuit. This was further connected to a strain gauge amplifier with a high bandwidth of ∼2.5 MHz. In addition to the piezo‐resistive strain gauges used for displacement measurement, a laser interferometer was employed in this work to validate displacements measured via the strain gauges. The laser interferometer, sourced from SmarAct Metrology (model V2 system), offers a displacement resolution of 1 pm and a measurement bandwidth of 2.5 MHz. A schematic of the displacement measurement setup is shown in Supplementary Figure S9B. In this configuration, the sample is mounted on the piezoelectric tube actuator, while a laser with a wavelength of 1545 ± 15 nm and a fixed focal length of 10 ± 0.5 mm is positioned opposite the actuator. The mirror‐polished surfaces of the samples are sufficient to reflect the laser and facilitate accurate displacement measurements. Predefined voltage profiles, corresponding to specific constant indentation strain rates, were designed and employed for recording the displacement using the laser interferometer.

For experiments conducted at the highest strain rate, the duration for the entire loading and unloading segment is approximately 150 µs, necessitating a high data acquisition rate to capture non‐aliased signals. To achieve this, Rohde & Schwarz RTA 4000 oscilloscopes with a 5 GSamples/s acquisition rate were employed. During all the experiments, across various strain rates, a minimum of 50 000 data points were recorded for every test to ensure high data fidelity. The oscilloscope features four input channels and an additional trigger port for initiating data acquisition. The monitor signal from the high‐voltage amplifier was used as the trigger for all experiments. To smoothen the data, all acquired signals were processed using zero‐phase low‐pass filters prior to any further analysis. All data processing was implemented in a custom‐written program developed in LabVIEW.

#### 4.1.1 Methodology for Signal Acquisition and Synchronization

Predefined shaped input exponential profiles for indentation were generated by the controller and sent to a high‐speed high voltage amplifier, which amplifies the signal 20‐fold to drive the piezoelectric tube actuator. The sample, mounted on the piezoelectric tube actuator, was then brought into contact with the indenter tip (Berkovich or flat punch) attached to the piezoelectric load cell, to perform either indentation or compression experiments.

The displacement was measured using piezo‐resistive strain gauges attached to the piezoelectric tube actuator, and the load was recorded via the charges output from the piezoelectric load cell. Both displacement and load signals were fed into a strain gauge amplifier and a high‐impedance charge amplifier, respectively, and the amplified analog output signals were captured using the oscilloscope. Additionally, the amplified monitor voltage (output voltage/10) from the high‐speed high‐voltage amplifier was recorded during all experiments. This monitored voltage captured using the oscilloscopes served two functions: triggering the oscilloscope to begin data acquisition and aligning the signals from all sensors.

In total, seven signals were recorded during the in situ experiments: four electrodes of the piezoelectric load cell, one from the piezo‐resistive strain gauge, and two mirrored monitored voltage signals from the high‐speed amplifier (later used for synchronization using a custom‐written LabVIEW software). Due to the oscilloscope's four‐input port limit, two oscilloscopes were used to capture all signals. The detailed schematic of the test setup is shown in Supplementary Figure S9.

In this work, in addition to the piezo‐resistive strain gauges, displacement was also measured using a laser interferometer, with the experiments being conducted ex situ on an optical table. The use of a second displacement measurement system was necessitated by the observation that the strain gauges mounted on the actuator provided inaccurate displacement readings when compared to those from the laser interferometer at very high strain rates (10^5^ s^−1^), as demonstrated in Supplementary Figure S10A. Several factors may contribute to the underperformance of strain gauges at such high velocities, including the mounting configuration of the strain gauges (strain gauges measure locally whereas laser interferometer directly measures at sample position) and the amplification errors/oscillations from the operational amplifiers [61]. It is important to note, however, that the piezo‐resistive strain gauges perform reliably, with deviations only observed in the last 20 nm of displacement at strain rates up to 10^4^ s^−1^, as shown in Supplementary Figure S11. The signal quality from the strain gauges deteriorates only beyond this strain rate. Although the laser interferometer proved highly reliable for displacement measurements, it could not be incorporated into the in situ experimental setup due to the compact design of the micromechanical test system. Consequently, displacement measurements using the laser interferometer were conducted ex situ, as illustrated in Supplementary Figure S9B, where the lower portion of the micromechanical setup, including the actuator and sample, was mounted on an optical table for displacement recording. To maintain consistency across all strain rates (10^1^ to 10^5^ s^−1^), the displacement data recorded via the laser interferometer were used for generating all the load‐displacement curves reported here. The same predefined voltage profiles used during the in situ indentation experiments were used in the ex situ laser interferometer displacement measurement experiments. Since the monitored voltage from the high‐speed voltage amplifier was consistently recorded in the oscilloscopes, these signals were utilized to synchronize the load data from in situ experiments with the displacement data from ex situ experiments, using a custom LabVIEW based program.

It was also critical to check whether the presence of the sample, indenter tip, and the piezoelectric load cell reduces the piezoelectric tube actuator displacement during in situ experiments when compared to the ex situ experiments without resistance from the tip and load cell. This concern was mitigated by the fact that the actuator stiffness (∼19.14 × 10^6^ N/m) was at least two orders of magnitude higher than the sample stiffness (∼10 × 10^4^ – 25 × 10^4^ N/m). In other words, the micromechanical system operates as an intrinsic displacement‐controlled setup. To further validate that the displacement was not reduced with or without the sample, Supplementary Figure S10B compares the ex situ displacement measured by the laser interferometer and the in situ displacement recorded during an indentation experiment at 10^1^ s^−^^1^, showing a negligible difference of 13 ± 2 nm.

#### 4.1.2 Time Synchronization

Since the load and displacement signals are captured by different electronic systems (outlined in the previous section) with differing processing times, a time lag arises between the two signals. To quantify this lag, the diamond Berkovich indenter tip was manually plunged into the sample surface using the piezo based positioning stages (on all three materials of molybdenum, nanocrystalline nickel, and fused silica) until distinct load signals were detected. While keeping the tip in contact with the sample surface, a near step‐like voltage profile was applied to the piezotube actuator and the signals were collected using an oscilloscope in a highly magnified temporal window such that even minimal time lags as small as 0.5 µs can be captured, as shown in the inset of Supplementary Figure S12A. From this calibration, a delay of 4.98 ± 0.05 µs was calculated between the load and displacement signals, as illustrated in Supplementary Figure S12A. The displacement data was also recorded ex situ using a laser interferometer, following the same voltage profile used in situ for load measurement. Synchronization between the in situ load signal and ex situ displacement data was achieved using the monitor voltage, aligned through a custom LabVIEW based program. The interferometer's displacement signal showed a longer lag than the load signal, attributed to the extended processing time of the interferometer's FPGA compared to the high‐impedance charge amplifier used for the load cell. Consequently, the displacement signal trails the load signal, as seen in Supplementary Figure S12A. Supplementary Figure S13 illustrates the resulting errors and time lag effects on load‐displacement data, which become noticeable and propagate at strain rates above 10^3^ s^−1^.

#### 4.1.3 Time Constant

For high‐speed measurement systems, the time constant of a sensor is defined as the time for a sensor to reach about 63.5% (1 – 1/e) of its steady‐state step input [62]. It is influenced by both the sensor's internal properties and associated electronics, including amplifiers. In systems like piezoelectric load cells, a shorter time constant enables faster response to dynamic load changes, which is essential for high strain rate experiments where measurements must occur within microseconds. A longer time constant, conversely, can introduce signal delays and smooth out transient events, leading to inaccuracies. To determine the time constant (*t_c_*) of the piezoelectric load cell in this study, we conducted compression tests on silicon pillars (5 µm diameter, 15 µm height), which are brittle and exhibit a sharp fracture. As seen in Supplementary Figure S12B, the load vs time data showed an exponential decay after fracture, rather than an instantaneous drop marked by a dotted black line, allowing us to fit this data to find a time constant of 1.70 ± 0.03 µs. Supplementary Figure S14 highlights the underestimation of load at higher strain rates when the time constant is not considered, with errors appearing above 10^3^ s^−1^. In this work, displacement measurements were captured using a laser interferometer with near‐instantaneous recording capabilities, eliminating the need for time constant correction for displacement data.

While time synchronization and time constant effects mainly impact data at strain rates above 10^3^ s^−1^, corrections were applied to lower strain rate data as well to ensure consistency across all analyses. Supplementary Figure S15 displays the load‐displacement curves for all materials tested in this study after applying time synchronization, time constant correction, compliance correction, and zero‐displacement correction. The zero‐displacement correction was determined via the Hertzian contact method to accurately establish the point of load initiation.

#### 4.1.4 Machine Dynamics

In nanoindentation experiments aimed at achieving constant strain rates, the input displacement profiles are typically designed as exponential functions of time. However, upon double differentiation of these exponential profiles with respect to time, it is clear that the acceleration as a function of depth is also not constant, and it continuously varies throughout the experiment. This varying acceleration introduces challenges due to the inertial forces that arise, as dictated by Newton's second law, whereby an increase in acceleration leads to a proportional increase in inertial force (*F = ma*). As strain rates increase, the duration of the exponential displacement profile becomes shorter, resulting in acceleration changes over shorter periods of time. Consequently, the inertial forces acting on the components of the nanoindentation system (such as the load cell) become more pronounced at higher strain rates. Inertial forces can distort the measured load data by introducing artifacts. As shown in Supplementary Figure S12C, the piezoelectric load cell and the piezoelectric tube actuator are arranged in series, with the load cell carrying its mass and movement in response to the external force applied by the actuator via the sample. Therefore, the inertial forces from the load cell must be accounted for when processing the raw load data. To calculate these forces, the system was modeled as a single‐degree‐of‐freedom damped harmonic oscillator. The piezoelectric load cell's response to the external force (*F*), was modelled using a Kelvin‐Voigt system. This model includes a viscous dashpot with a damping constant *b* = 10.217 Ns/m and an elastic spring with stiffness *k* = 32.208 × 10^6^ N/m, arranged in parallel with a mass of *m* = 0.238 g, as illustrated in Supplementary Figure S12C. Details of the calculations for each parameter, along with a comprehensive methodology and error analysis for the machine dynamics corrections applied in this study, are provided in detail in – Supplementary information Section S2 (Machine dynamics – Methodology and error analysis). Supplementary Figure S12D shows the loads attributable to machine dynamics across various strain rates for molybdenum, as determined by the machine dynamics model. At the highest strain rate, machine dynamics account for a reduction of approximately 16.8 ± 1.6 µN in force at around 700 nm of displacement, equating to roughly 6% of the actual load on the sample. The dynamic load contribution varies based on the force acting on the piezoelectric load cell, and the calculated dynamic loads for fused silica and nanocrystalline nickel are presented in Supplementary Figure S16. These figures indicate that the load contribution due to machine dynamics becomes significant only at strain rates 10^4^ s^−1^ and beyond for this specific system configuration. Supplementary Figure S12E provides a perspective on the cumulative effect of all three corrections—time synchronization, time constant, and machine dynamics—applied to an experiment conducted on molybdenum at a strain rate of 10^5^ s^−1^.

The detailed methodology of nanoindentation experiments, micropillar compression testing, quasi‐static reloading experiments, hardness measurement, and TEM, along with dislocation density measurement, is given in – Supplementary Information Section S3.

## Author Contributions

L.K.B., G.D., and R.R. contributed to the conceptualization of the study. L.K.B. and R.R. contributed to the methodology. All authors were involved in the investigation. G.D. and R.R. contributed to funding acquisition. R.R. provided supervision. L.K.B. prepared the original draft of the manuscript. All authors contributed to writing through review and editing.

## Funding

L.K.B., J.P. and R.R. would like to acknowledge funding from the European Research Council (ERC) (Starting grant agreement No. 101 078 619; AMMicro). D.S. and R.R. would like to acknowledge funding from the Eurostars Project HINT (01QE2146C). D.S. and B.B. would like to acknowledge funding from the Alexander von Humboldt Foundation, and B.B., in addition, would like to acknowledge funding from the Marie Sklodowska‐Curie individual fellowship (Grant agreement No. 101 064 660; DyThM‐FCC). Views and opinions expressed are however those of the author(s) only and do not necessarily reflect those of the European Union or the European Research Council. Neither the European Union nor the granting authority can be held responsible for them.

## Conflicts of Interest

The authors declare no competing interests.

## Supporting information




**Supporting File**: smll73215‐sup‐0001‐SuppMat.pdf.

## Data Availability

All data are available in the main manuscript or the supplementary materials.
