# Supplementary material for: Filling a Gap in Materials Mechanics: Nanoindentation at High Constant Strain Rates up to 105 s−1
Source: Small. 2026 Apr 1;22(28):e73215. doi: 10.1002/smll.73215 (PMC13181535; doi:10.1002/smll.73215)
Supplement: Supplementary file 1 — Supporting File: smll73215‐sup‐0001‐SuppMat.pdf. [file SMLL-22-e73215-s001.pdf]

Supplementary information

Filling a gap in materials mechanics: Nanoindentation at high constant strain rates up to 10^5 s^{-1}

Lalith Kumar Bhaskar*[1], Dipali Sonawane[1], Hendrik Holz[1], Jeongin Paeng[1], Peter Schweizer[1], Jing Rao[1], Bárbara Bellón[1], Damian Frey[2], Aloshious Lambai[3,4], Laszlo Pethö[5], Johann Michler[5,6], Jakob Schwiedrzik[7], Gaurav Mohanty[3], Gerhard Dehm*[1], Rajaprakash Ramachandramoorthy*[1]

[1] Max-Planck-Institute for Sustainable Materials, Department of Structure and Micro-/Nano- Mechanics of Materials, Max Planck-Strasse 1, 40237 Düsseldorf, Germany

[2] Alemnis AG, Schorenstrasse 39, 3645 Thun, Switzerland

[3] Materials Science and Environmental Engineering, Faculty of Engineering and Natural Sciences, Tampere University, 33014 Tampere, Finland

[4] Current address: VTT Technical Research Centre of Finland Ltd., Kemistintie 3, FI-02044 Espoo, Finland

[5] Laboratory of Mechanics of Materials and Nanostructures, Empa – Swiss Federal Laboratories for Materials Science and Technology, Feuerwerkerstrasse 39, 3602 Thun, Switzerland

[6] EPFL, Ecole Polytechnique Fédérale de Lausanne, Institute of Materials (IMX), CH-1015 Lausanne, Switzerland

[7] Laboratory for High Performance Ceramics, Empa – Swiss Federal Laboratories for Materials Science and Technology, Ueberlandstrasse 129, 8600 Dübendorf, Switzerland

*Corresponding author: l.bhaskar@mpi-susmat.de; g.dehm@mpi-susmat.de; r.ram@mpi-susmat.de

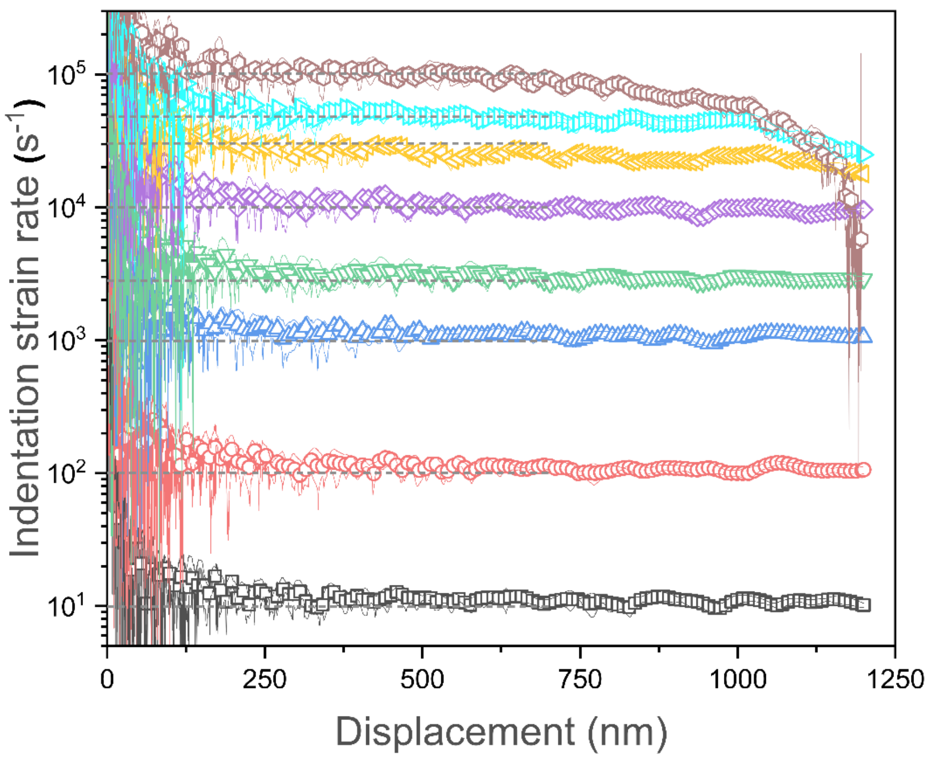

Fig. S1.

The strain rate variations as a function of indentation depth.

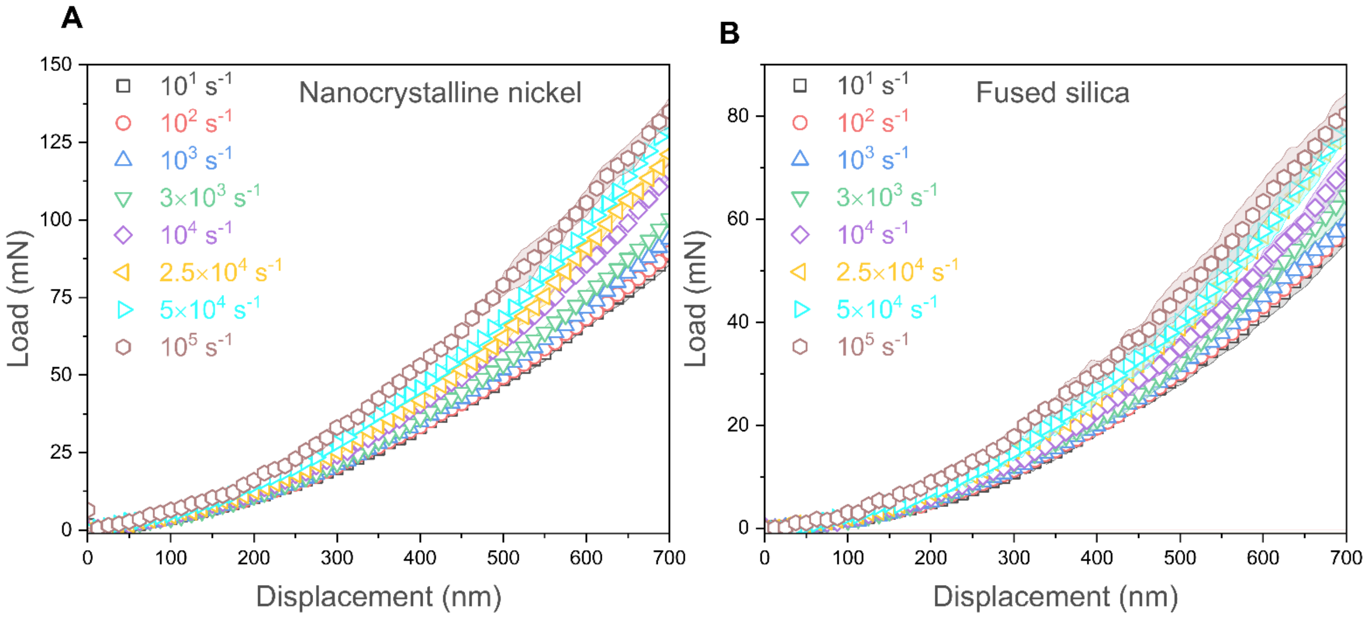

Fig. S2.

The load-displacement curve corresponding to the loading segment for (**A**) nanocrystalline nickel and (**B**) amorphous fused silica after applying all correction protocols. The curves are a result of average of five experiments carried out at each strain rate.

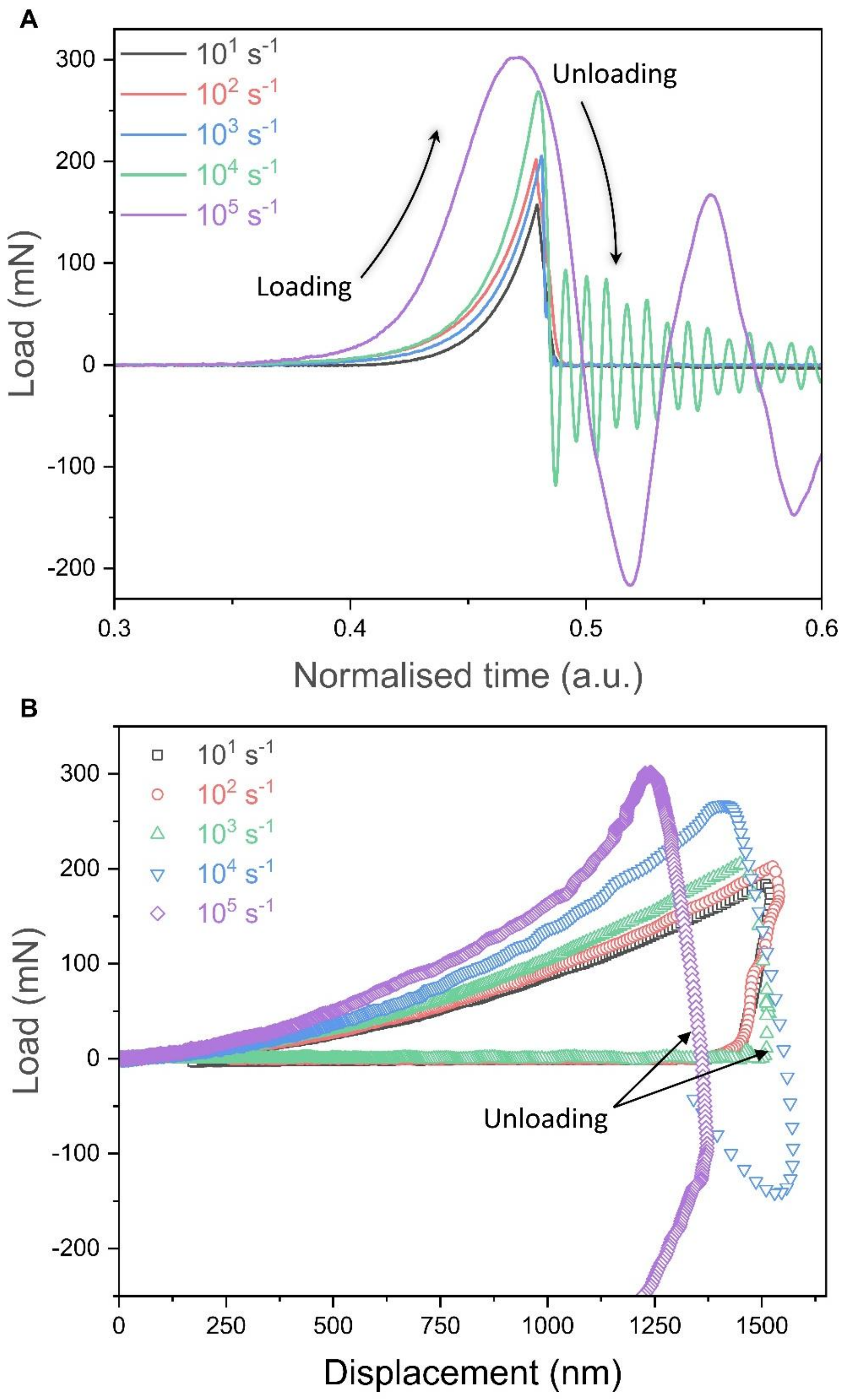

Fig. S3.

(A) Representative experimental load-normalised time curves and it can be observed that unloading segments starting from 10^3 s^{-1} are affected by resonance from various components in the testing platform. **(B)** Shows the representative unloading curves for molybdenum at different strain rates, after applying all corrections

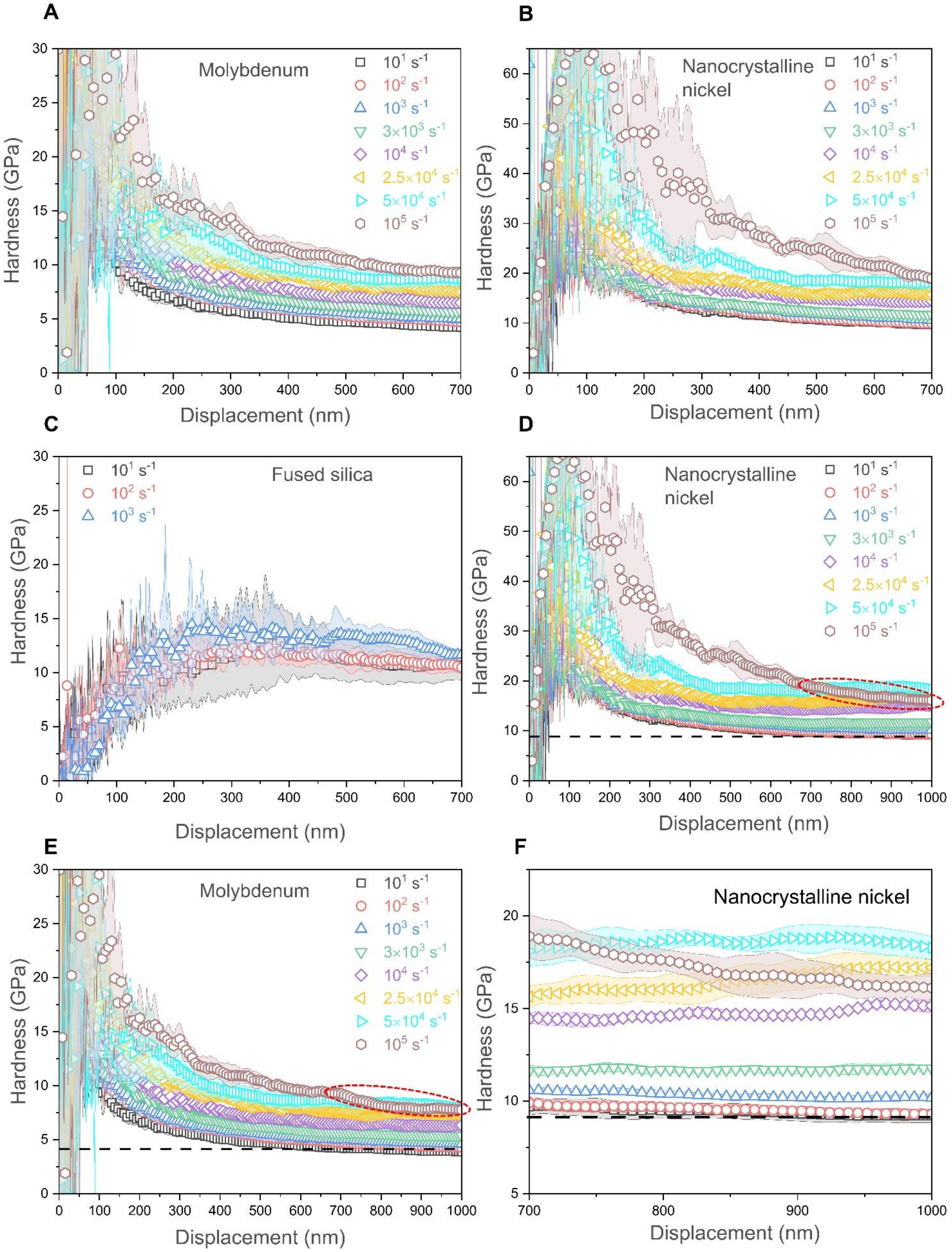
A
Molybdenum
Hardness (GPa)
Displacement (nm)
10^1 s^-1
10^2 s^-1
10^3 s^-1
3×10^3 s^-1
10^4 s^-1
2.5×10^4 s^-1
5×10^4 s^-1
10^5 s^-1
B
Nanocrystalline nickel
Hardness (GPa)
Displacement (nm)
C
Fused silica
Hardness (GPa)
Displacement (nm)
D
Nanocrystalline nickel
Hardness (GPa)
Displacement (nm)
E
Molybdenum
Hardness (GPa)
Displacement (nm)
F
Nanocrystalline nickel
Hardness (GPa)
Displacement (nm)

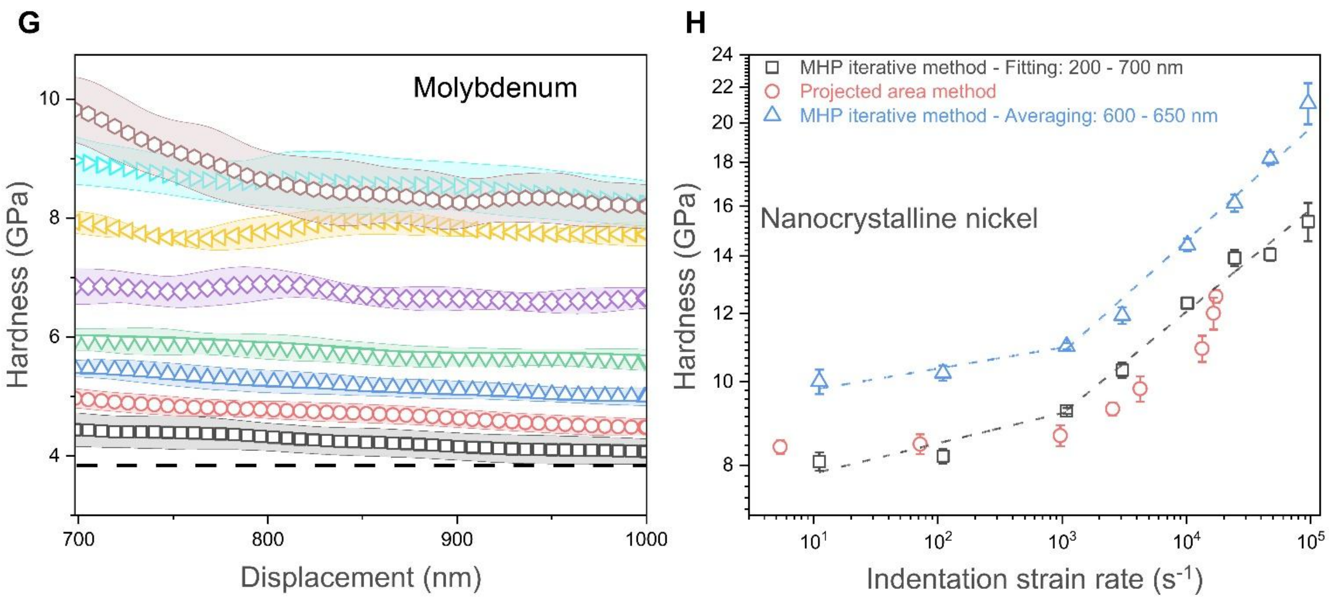

Fig. S4.

Hardness vs. displacement plots for **(A)** molybdenum, **(B)** nanocrystalline nickel, and **(C)** fused silica obtained using the MHP iterative method. Each data point includes error bars representing the standard deviation from five independent measurements. Hardness vs. displacement for **(D)** nanocrystalline nickel and **(E)** molybdenum up to a depth of 1000 nm. The decrease in hardness due to decrease in strain rate beyond 700 nm at a strain rate of 10^5 s^{-1} is highlighted with a dotted red ellipse. Enlarged view of Hardness vs. Displacement plots for **(F)** nanocrystalline nickel and **(G)** molybdenum at depths greater than 700 nm. The results show that hardness has already saturated for nanocrystalline nickel, whereas molybdenum continues to exhibit an indentation size effect. A dotted horizontal line is included as a reference to highlight the saturation behaviour. **(H)** Comparison of nanocrystalline nickel hardness values determined by three methods: averaging over 600–650 nm, Nix–Gao fitting and extrapolation (200–700 nm), and the projected area method.

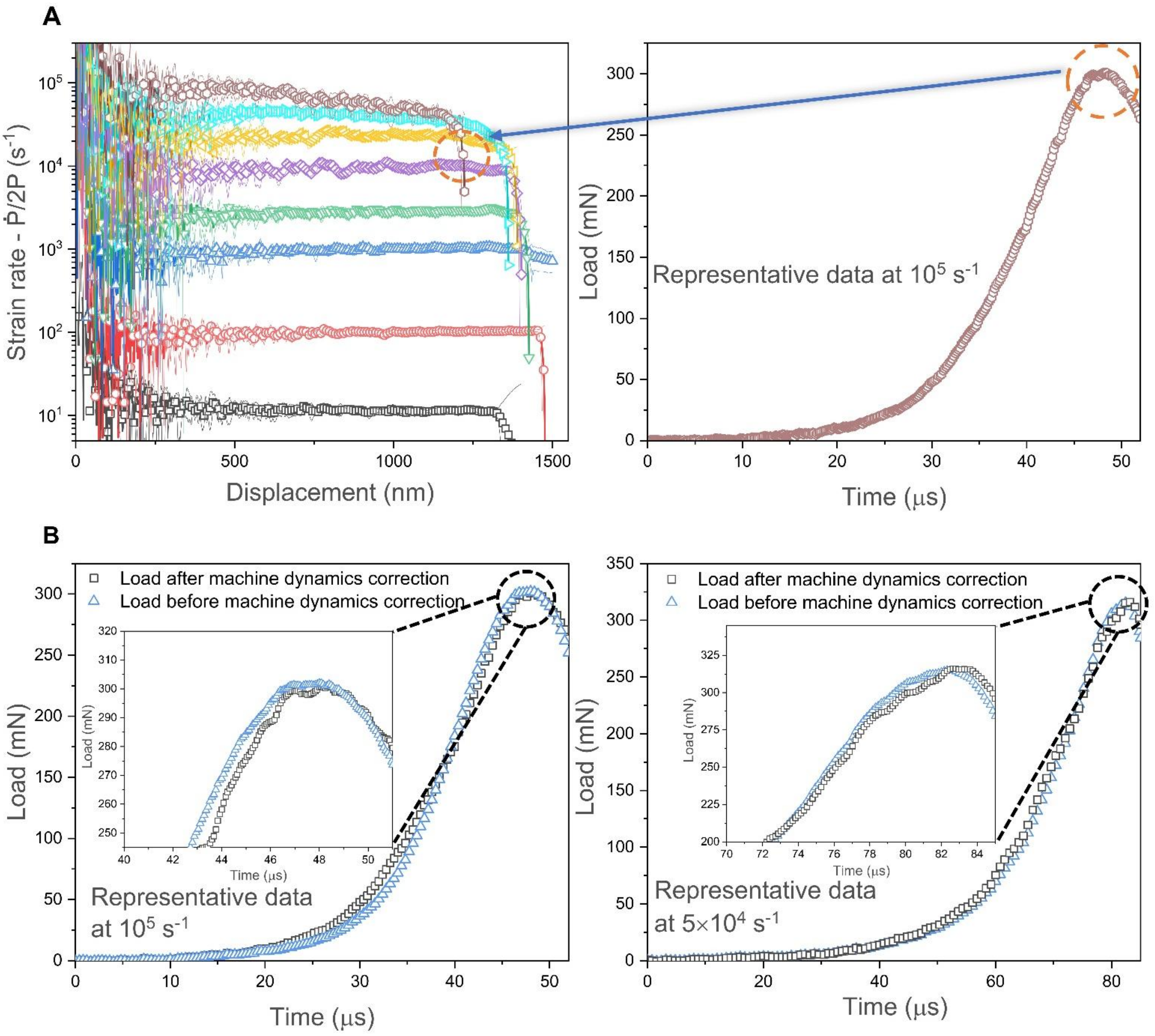

Fig. S5.

(**A**) At the highest load, the strain rate falls below the expected/prescribed strain rate, and (**B**) for representative data at 10^5 s^{-1} and 5×10^4 s^{-1}, the peak load value remains unchanged after applying all corrections despite changes observed in the loading portion of the curve. Similar trends are observed across all strain rates.

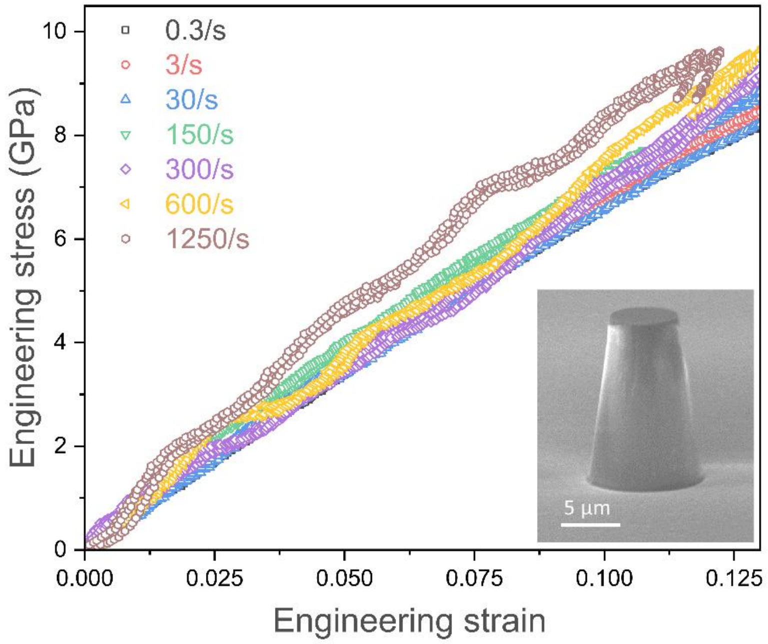

Fig. S6.

Shows the engineering stress–strain curves for fused silica micropillars of 14.5 µm height tested at strain rates ranging from 0.3 s^{-1} to 1250 s^{-1}. The inset figure shows a representative 14.5 µm high fused silica pillar before compression.

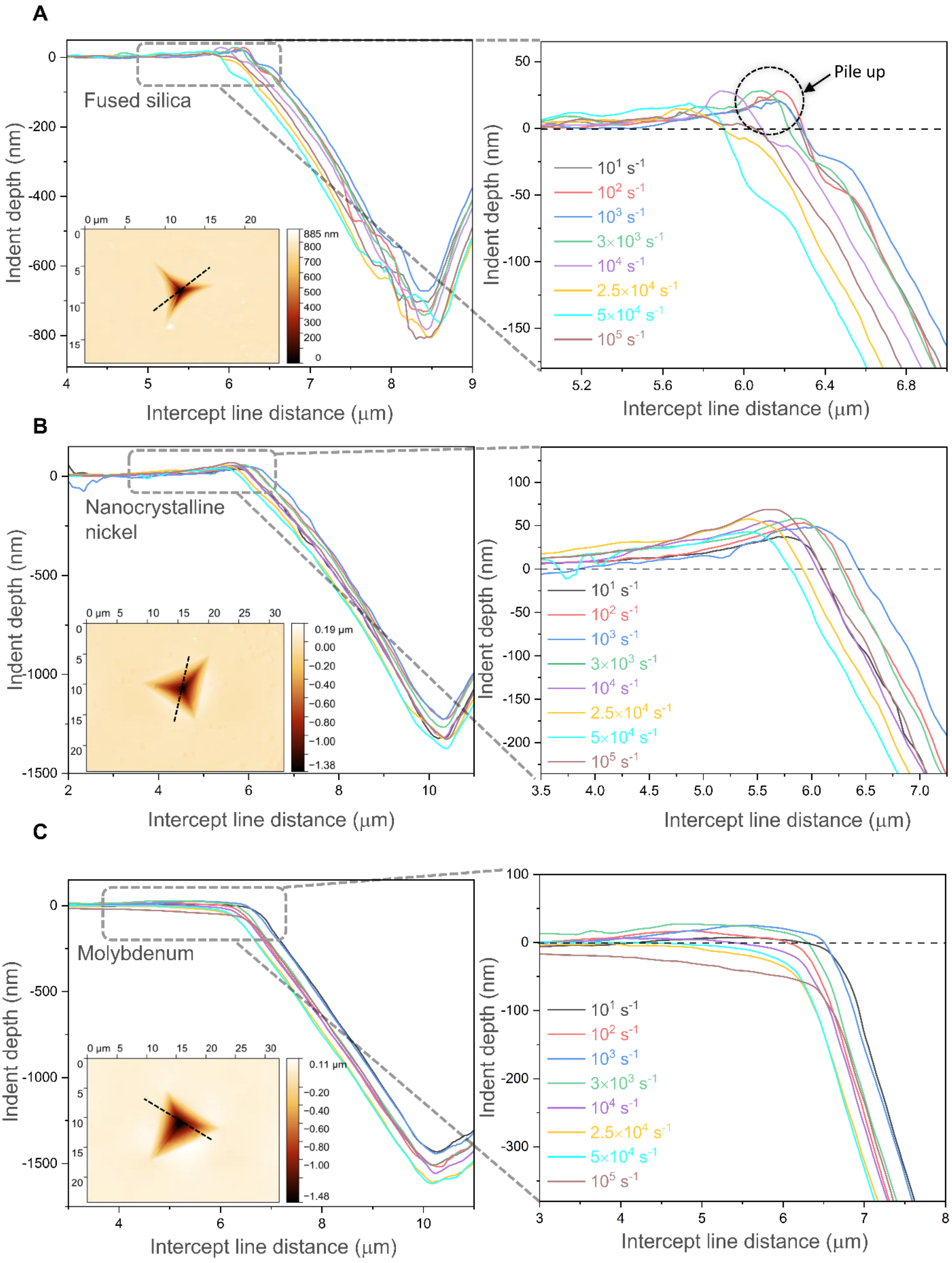

Fig. S7.

Cross-sectional line profiles of the indents parallel to one of the indent edges for (**A**) fused silica, (**B**) nanocrystalline nickel and (**C**) molybdenum. The inset figure shows a representative confocal image with the direction along which line profiles were taken.

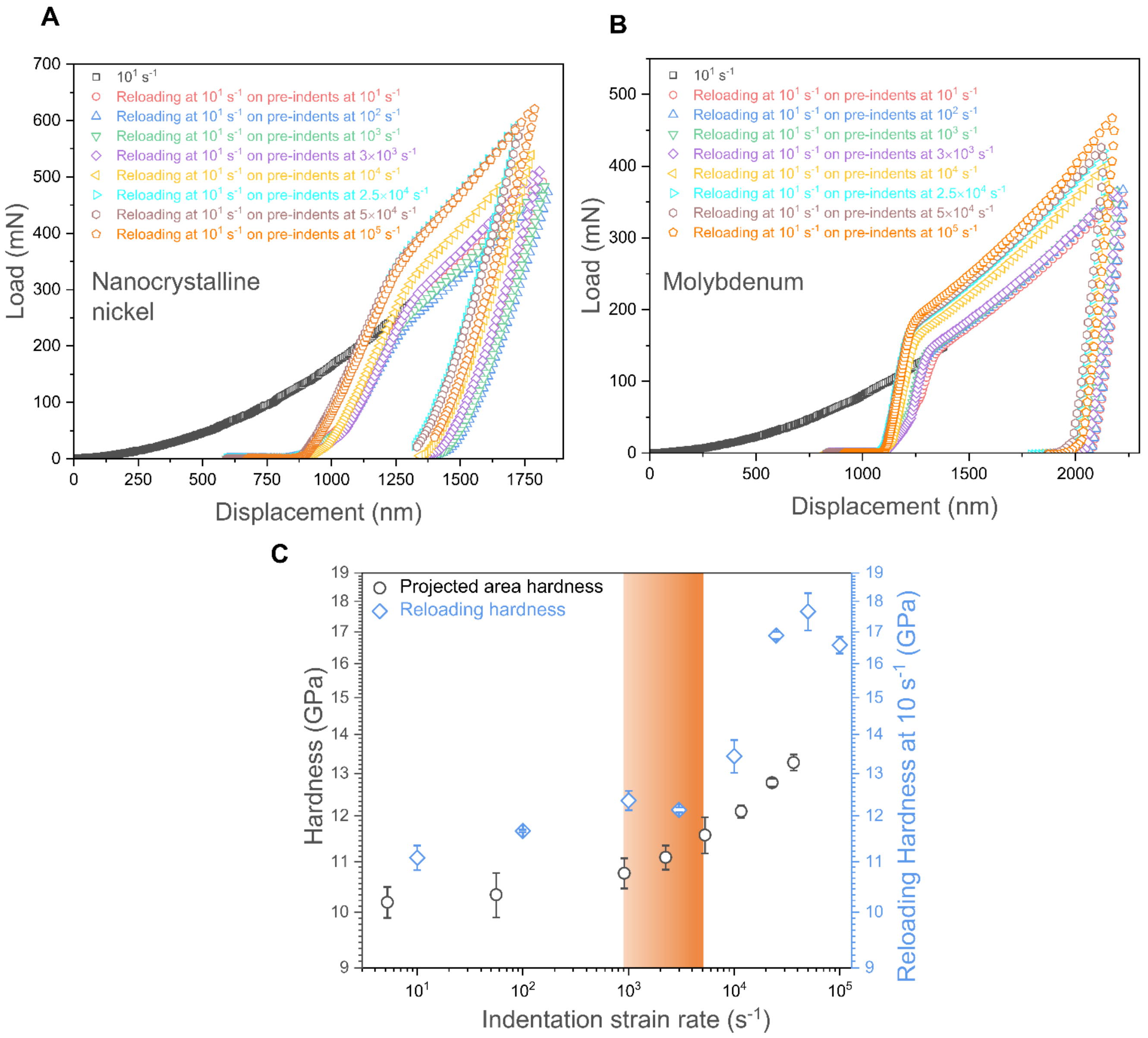

Fig. S8.

Reloading load-displacement curves at 10^1 s^{-1} on pre-existing indents created at various strain rates for (**A**) nanocrystalline nickel and (**B**) molybdenum and (**C**) reloading hardness trends for fused silica, with hardness vs. strain rate trends overlaid for clarity. The strain rate at which the hardness upturn is observed is marked by an orange band and the hardness values are in log scale

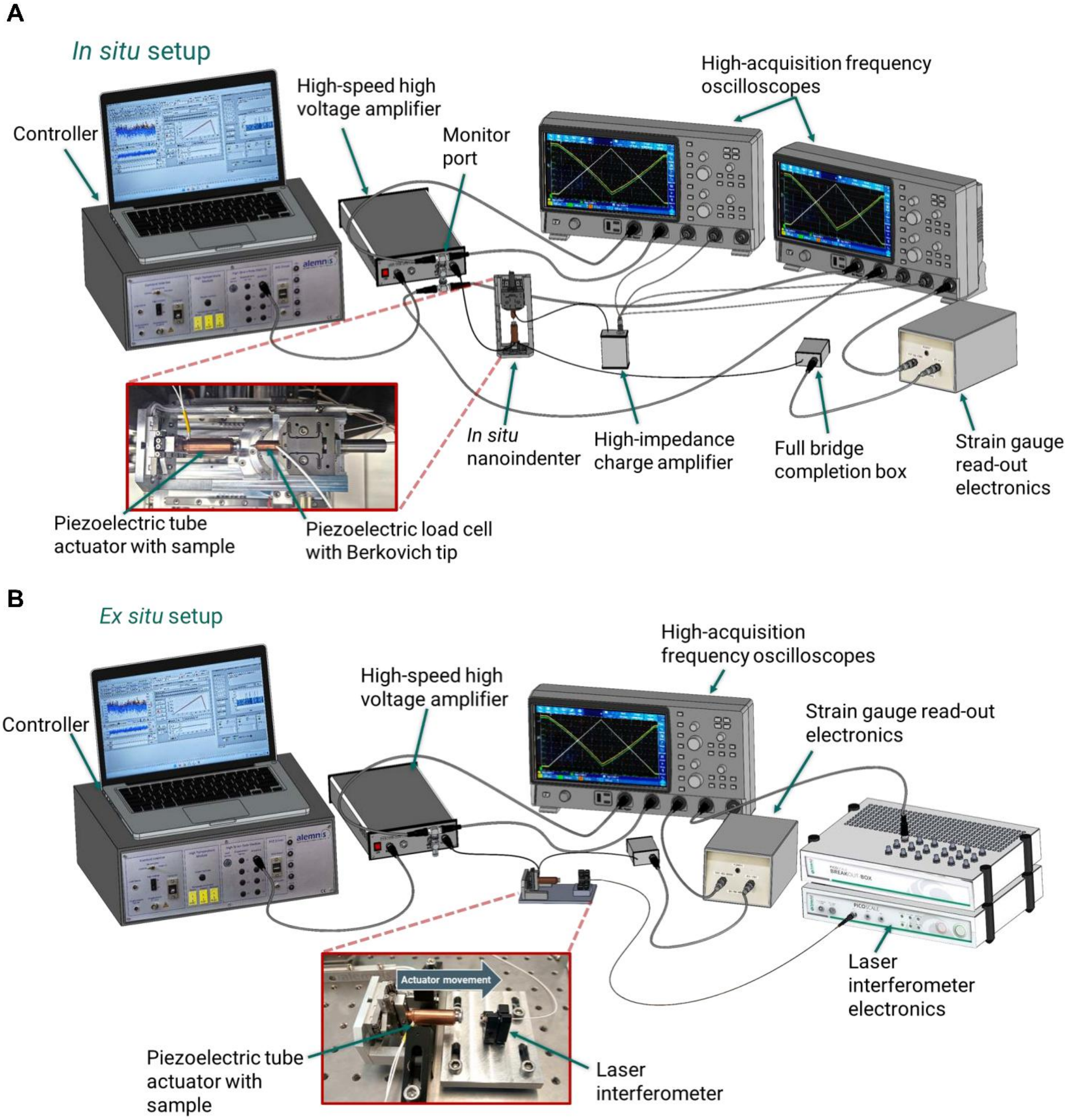

Fig. S9.

The (**A**) *in situ* and (**B**) *ex situ* setups with the various components used for performing high constant strain rate indentations.

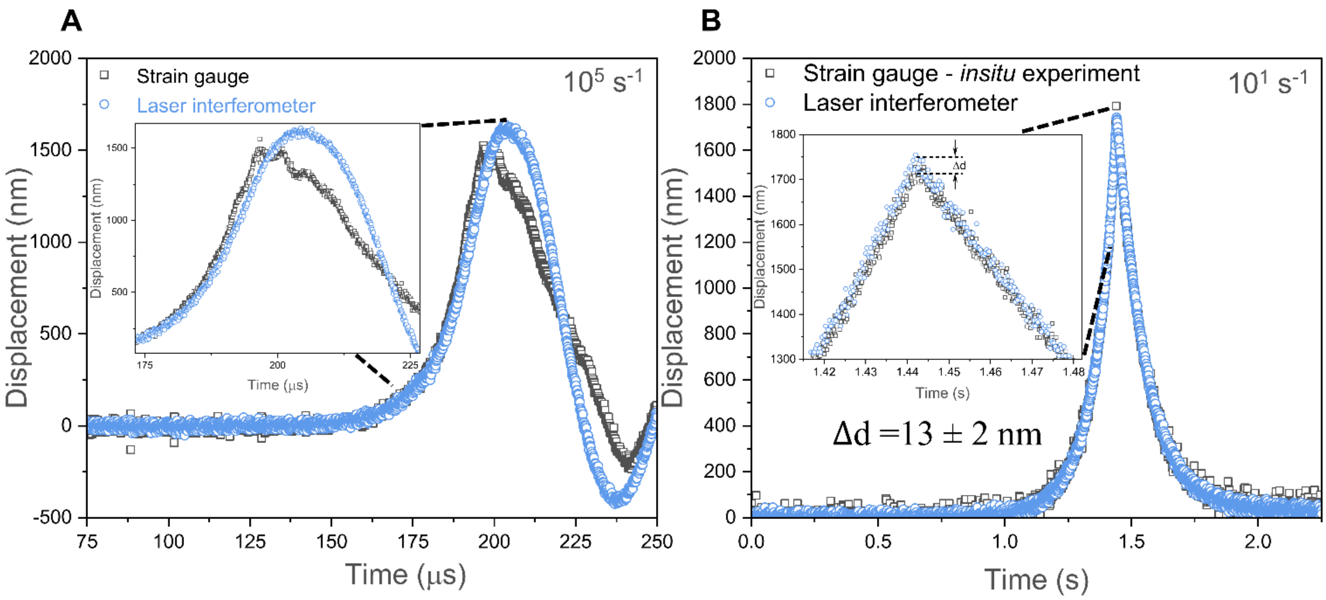

Fig. S10.

(**A**) A comparison illustrating that strain gauges mounted on the actuator provide inaccurate displacement readings at very high strain rates (10^5 s^{-1}) when compared to the laser interferometer; and (**B**) a comparison between *ex situ* displacement measurements from the laser interferometer and *in situ* displacement data recorded during an indentation experiment at 10^1 s^{-1}, showing little to no variation in displacements.

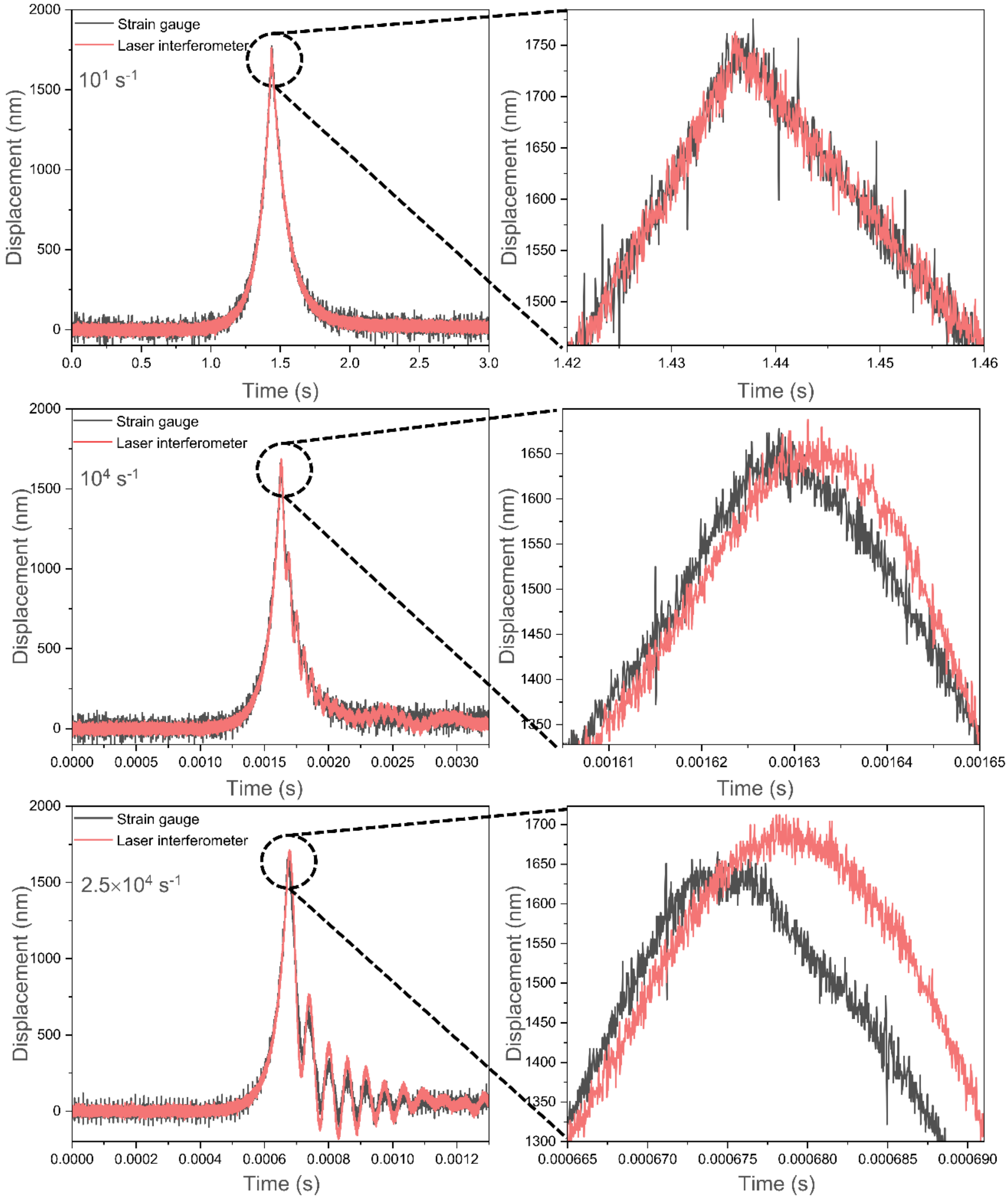

Fig. S11.

The displacement data recorded using both the strain gauge and laser interferometer across various strain rates. It is evident that at strain rates exceeding 10^4 s^{-1}, the piezo-resistive strain gauges mounted on the actuator fail to provide accurate data.

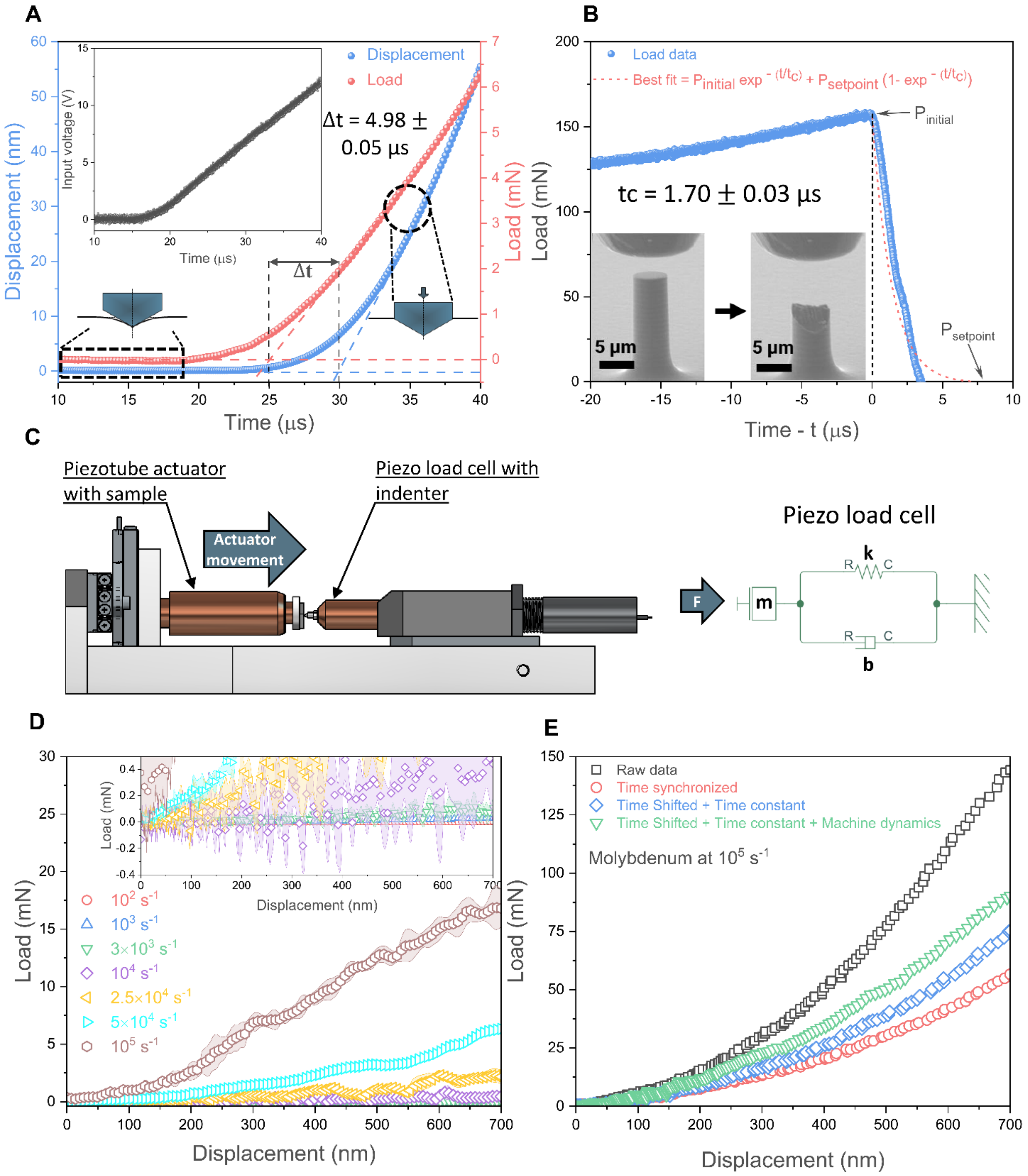

Fig. S12.

(**A**) The time lag between load and displacement signals, with inset showing the sharp temporal voltage profile sent to the actuator to capture this lag, and a schematic of the test process. (**B**) Load versus time data for silicon pillar fracture, showing an exponential decay post-fracture rather than an immediate drop (dotted black line); time constant (t_c) is calculated using the equation in legend. (**C**) Schematic arrangement of series arrangement of the piezoelectric load cell and piezoelectric tube actuator, with the equivalent Kelvin-Voigt model of the piezoelectric load cell under external force. (**D**) Load contributions from machine dynamics at various strain rates for molybdenum with the inset showing for lower strain rates. (**E**) The cumulative effect of corrections—time synchronization, time constant, and machine dynamics.

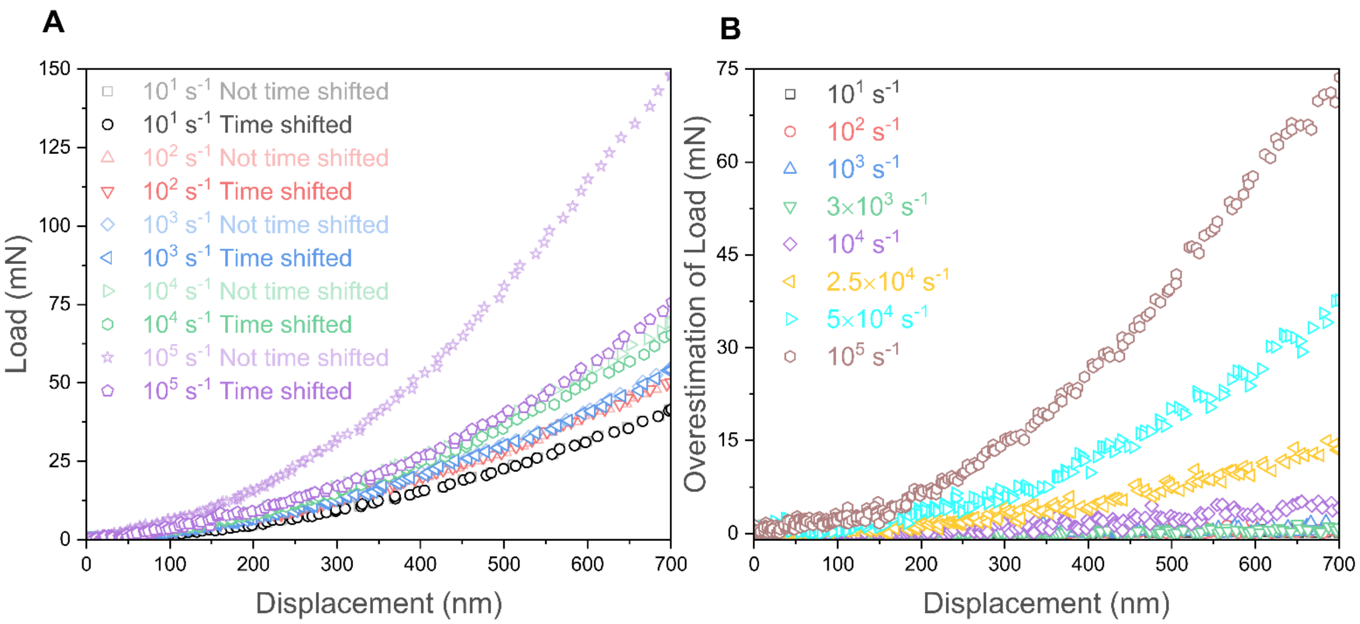

Fig. S13.

(**A**) The effect of the 4.98 ± 0.05 μs time lag on the load-displacement data for molybdenum at different strain rates, while (**B**) highlights the load overestimation as a function of displacement across all tested strain rates if time synchronization is not applied. At strain rates exceeding 10^3 s^{-1}, the error becomes noticeable and starts to propagate due to the time lag.

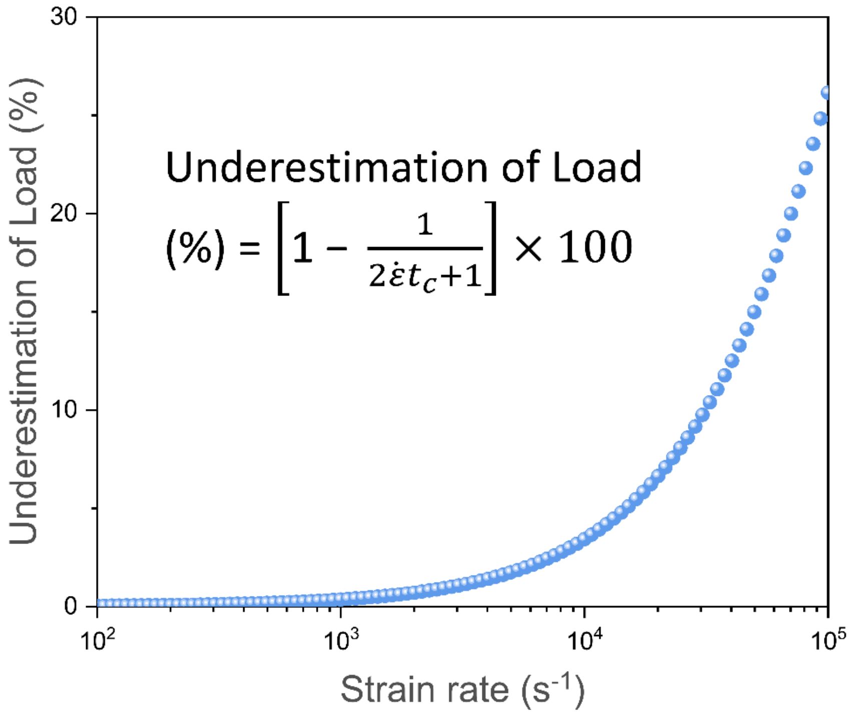

Fig. S14.

The percentage underestimation of the load signal as a function of strain rate when the time constant (t_c) is not considered.

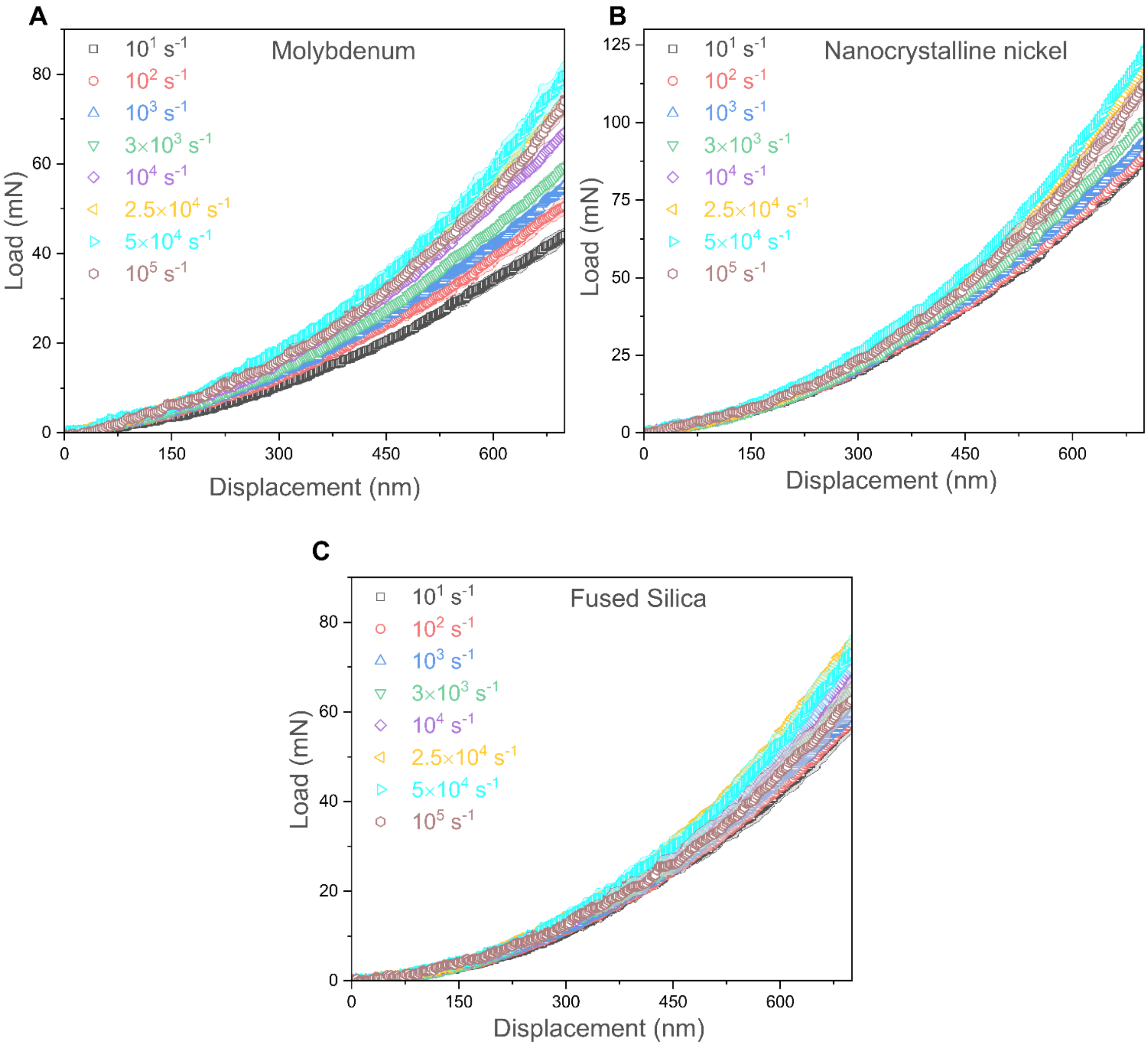

Fig. S15.

The load-displacement curves after applying time synchronization, time constant correction, compliance correction, and zero-displacement correction for (**A**) molybdenum, (**B**) nanocrystalline nickel, and (**C**) fused silica.

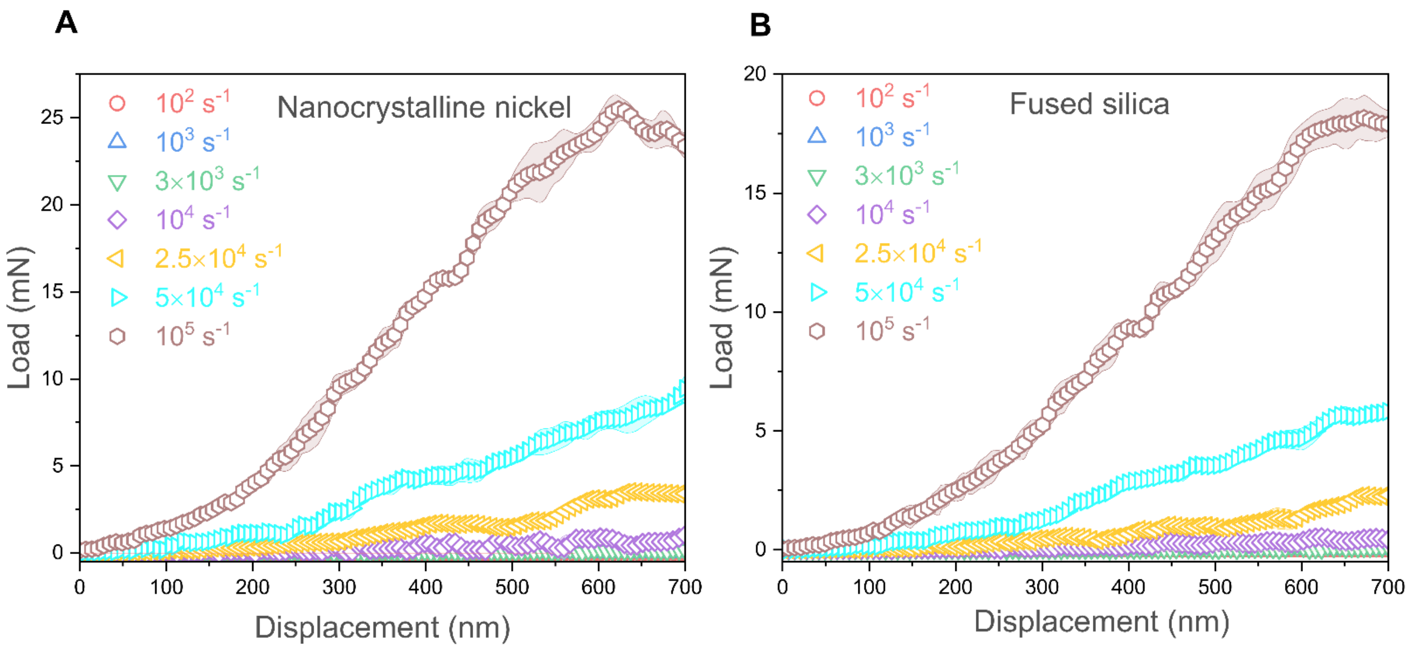

Fig. S16.

The load contributions from machine dynamics at various strain rates for (**A**) nanocrystalline nickel and (**B**) fused silica.

S1 Strengthening mechanism

S1.1 Lattice resistance strengthening

In most polycrystalline materials, the motion of a dislocation is constrained by its interaction with the atomic structure known as the Peierls force or lattice resistance, this arises from fluctuations in the dislocation's energy as it moves through the crystal lattice. The magnitude and periodicity of these fluctuations are influenced by the strength and spacing of the interatomic or intermolecular bonds. It is given by equation,

$$\sigma_l = \left[1 - \left(\frac{kT}{g_0 \mu_0 b^3} ln\left(\frac{\dot{\varepsilon}_0}{\dot{\varepsilon}}\right)\right)^{1/q}\right]^{1/p} \sigma_0 \qquad (1)$$

Here, g_0 is a constant related to the strength of the obstacles, determined by the microstructure. In this case, it represents the lattice resistance with a value of 0.1. b is the magnitude of the Burgers vector, equal to 0.272 nm, and μ_0 is the shear modulus with a value of 134 GPa. The constants p and q are exponents that describe the shape and spacing of energy barriers associated with the obstacles, with values of 3/4 and 4/3, respectively. k represents Boltzmann's constant, T is the temperature (298 K in this case), σ_0 is the stress required to overcome the

relevant short-range barrier at 0 K, valued at 0.8844 GPa, and $\dot{\varepsilon_0}$ is the reference strain rate, set at 10^{11} s^{-1}. All constant values are sourced from Harold and Ashby, *Deformation of Mechanism Map* [1].

S1.2 Dislocation strengthening

In addition to lattice resistance, long-range obstacles like far-field forest dislocations in a single crystalline material can create a potential field that hinders dislocation motion. The interaction strengthening is given by the following Taylor equation,

$$\sigma_{disl} = \alpha_{disl}\mu_0 b\sqrt{\rho_t} \qquad (2)$$

Here, α_{disl} is the dislocation-dislocation interaction parameter and is equal to 0.5 and ρ_t is the total dislocation density in the sample and is obtained from the TEM analysis.

S1.3 Dislocation-Phonon drag strengthening

When the applied stress is sufficiently high, dislocations can bypass obstacles continuously, accelerating to high steady-state velocities, where dislocation interacts with, phonons and electrons and which give rise to strengthening. The increase in strength due to phonon drag is given by the equation,

$$\sigma_d = \frac{B_d}{\rho_m b^2}\dot{\varepsilon} \qquad (3)$$

$$B_d = \frac{3kT}{5 \cdot Ca^3}(1.8b) \qquad (4)$$

Here, ρ_m represents the mobile dislocation density, which is typically a fraction of the total dislocation density [2] . For molybdenum, a BCC material with higher lattice resistance, ρ_m is assumed to be, $\rho_m = 10^{-3} \times \rho_t$. B_d is the damping constant in which, C denotes the shear wave velocity, which for molybdenum is 3500 m/s and a is the lattice parameter with a value of 3.147 Å and k represents Boltzmann's constant, T is the temperature (298 K in this case) and b is the magnitude of the Burgers vector, equal to 0.272 nm. Further details on this formulation can be found in I. Dowding *et al.* [3].

S2 Machine dynamics – Methodology and error analysis

As the strain rate increases, it is clear that the force applied on the piezoelectric load cell is fast enough to encounter inertial resistance. Due to this inertial resistance, the compression of the load cell is smaller than expected, meaning the charges output and consequently the measured

load ($P_{measured}$) is underestimated compared to the actual true load (P_{true}). Hence, to obtain the true load, the measured load must be compensated appropriately with the loads that were lost due to the inertial resistance, as given in Equation 5 below:

$$P_{true} = P_{measured} - (-m\ddot{x} - b\dot{x}) \qquad (5)$$

Here, $\ddot{x}$ and $\ddot{x}$ represents the acceleration and velocity of the piezoelectric load cell respectively. In order to calculate the acceleration and velocity one needs to determine the compressive displacement (x) of the piezoelectric load cell due to external force (F) acting on the load cell. From classical mechanics, internal second body compressive displacement (x) of the piezoelectric load cell can be related to external force through the following relation,

$$F = kx \qquad (6)$$

In the literature [4], for systems with similar configurations, a common simplification to evaluate the compressive displacement (x) has been to assume that the measured load ($P_{measured}$) is same as the external force (F). However, it is clear that the inertial resistance of the load cells does influence the measured load, which would affect the calculated internal compressive displacement (x) of the load cell. Therefore, in this study, a different approach was implemented by using a *mock load* (F_{mock}) rather than the measured load ($P_{measured}$) to obtain the compressive displacement of the load cell and consequently the required compensation to obtain the true load (P_{true}) accurately.

However, before determining the internal compressive displacement (x) of the second body (piezoelectric load cell), several other variables must be identified, including the damping coefficient (b), elastic spring stiffness (k), and mass (m). To determine the total mass, the masses of the piezo element in the load cell, the diamond Berkovich tip, and the connector interface between the indenter and the piezo element must be identified. Each component was individually weighed in a precision balance: the mass of the diamond Berkovich tip = 0.10 ± 0.01 g, the connector interface = 0.05 g (estimated from CAD model) and the piezo element of the load cell = 0.265 ± 0.005 g. However, from classical mechanics, when a mass undergoes oscillation or rotation about one end, its contribution to the system's inertia can be approximated by concentrating a fraction of the total mass at a specific location—typically one-third of the mass in cases resembling a cantilevered beam[5]. This simplification captures the dynamic effect of the distributed mass by representing it as a point mass located at an equivalent position, determined by the system's geometry and mode of vibration. In this study, only the piezo element of the load cell is actively oscillating. Therefore, its equivalent mass is

considered in the dynamic analysis. This is justified by the fact that, for the fundamental bending mode of a uniform cantilever beam, the effective mass at the free end is well-established to be one-third of the total mass and is given by, $m_{eff} = \frac{1}{3}m$. This approximation effectively accounts for the portion of the piezo's mass that contributes to dynamic motion.

Therefore, the total mass (m) is calculated as follows:

$$Total\ mass\ (m) = \frac{1}{3}\ mass\ of\ piezo\ element + mass\ of\ connector\ interface + mass\ of\ diamond\ Berkovich\ tip \qquad (7)$$

$$\therefore m = \frac{0.265 \pm 0.005}{3} + 0.05 + (0.10 \pm\ 0.01) = 0.238\ \pm\ 0.010\ \text{g}$$

The stiffness (k) of a cylinder can be estimated by the following equation,

$$Stiffness\ (k) = \frac{EA}{L} \qquad (8)$$

Here, the elastic modulus (E) of the piezoelectric cylinder used in the load cell was obtained from the supplier (PI Ceramic GmbH). The stiffness matrix of the piezo as given by the supplier is,

C_{11} = 133.2 GPa

C_{12} = 87.15 GPa

C_{13} = 86.19 GPa

C_{33} = 120 GPa

C_{44} = 21.76 GPa

C_{55} = 21.76 GPa

C_{66} = 23.02 GPa

Then using the ELATE tool [6] the elastic modulus (E) was calculated to be 60.753 GPa.

The area (A) of the piezoelectric tube is given by,

$$Area\ (A) = \frac{\pi}{4}(Outer\ diameter^2\ -\ Inner\ diameter^2)$$

$$= \frac{\pi}{4}((3.2E-3)^2\ -\ (2.2E-3)^2) = 4.24E-6\ m^2 \qquad (9)$$

And the length of the tube (L) was measured to be 8mm. Substituting all the values in equation (6) the stiffness (k) was calculated to be 3.22E7 N/m.

The damping coefficient (b) of the system was determined by calculating the damping ratio (ξ) of the piezoelectric load cell. For this, the free body oscillation of the load cell after a quick excitation was collected. Fig. S17 shows the free body oscillation of the piezoelectric load cell from three different experiments after an indentation experiment which was carried out at 5×10^4 s^{-1}. Also, shown is the exponential fit to estimate the logarithmic decay (δ). The damping ratio (ξ) is given by the following relation,

$$\xi = \frac{b}{C_c} \qquad (10)$$

Where C_c is the critical damping coefficient and is given by,

$$C_c = 2\sqrt{k \times m} \qquad (11)$$

Substituting for stiffness (k) and mass (m) the critical damping coefficient (C_c) was calculated to be 175.2 $\frac{Ns}{m}$. The damping ratio (ξ) in turn is related to the logarithmic decrement (δ) by the following relation [5],

$$\xi = \frac{1}{\sqrt{(1 + \left(\frac{2\pi}{\delta}\right)^2}} \qquad (12)$$

And logarithmic decay (δ) in turn is given by,

$$\delta = \ln\frac{x_1}{x_2} \qquad (13)$$

where x_1 and x_2 are amplitudes of two successive oscillations as shown in fig. S17.

Using equations (10-13) the damping coefficient of the piezoelectric load cell was calculated to be 10.5 ± 5.5 $\frac{Ns}{m}$.

In this work, a novel approach was employed to determine the external force (cap F) on the load cell accurately. In this work, in total eight strain rates were tested - 10^1, 10^2, 10^3, 3×10^3, 10^4, 2.5×10^4, 5×10^4, and 10^5 s^{-1}. For the indentation experiment at 10^1 s^{-1}, which has a duration of about 0.5 seconds, the influence due to inertial resistance from the load cell is minimal, so no inertial correction was needed. For strain rates higher than 10^2 s^{-1} a correction owing to the

inertial resistance of the load cell was applied. The following procedure was followed to compensate for the inertial resistance of the load cell based underestimation of the true load at strain rates beyond 10^2 s^{-1}.

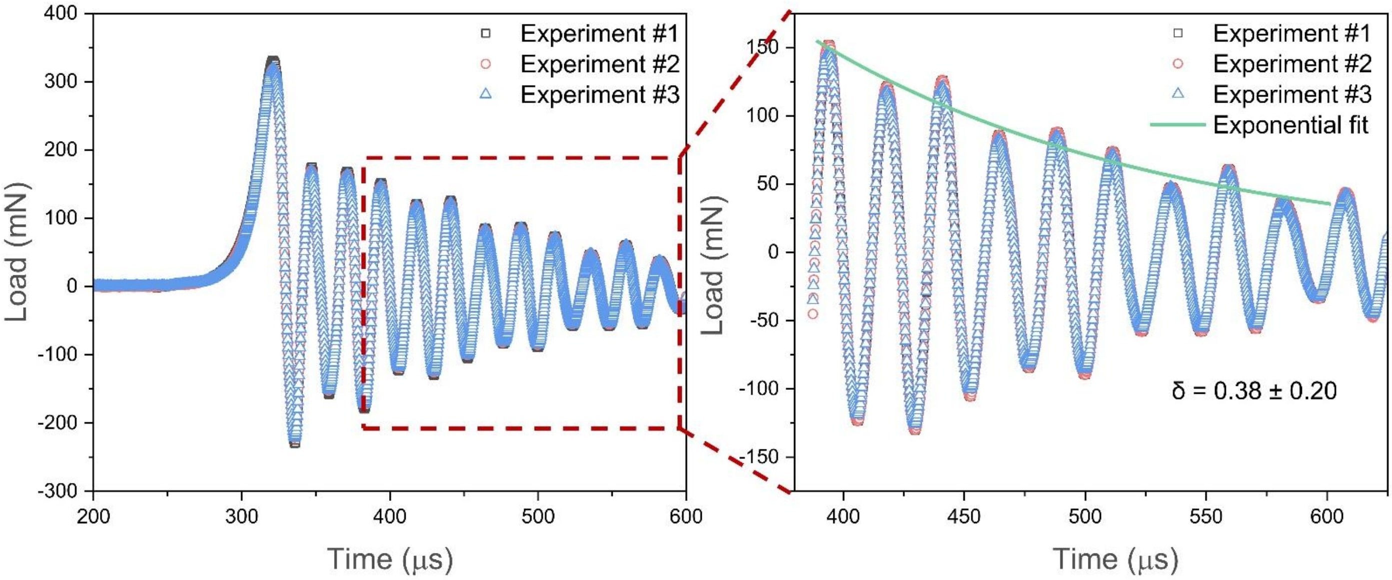

Fig. S17.

The free body oscillation of the piezoelectric load cell after an indentation experiment, which was carried out at 5×104 s^{-1} in molybdenum. For statistics, three loading curves are shown. Also, shown is the logarithmic fit to estimate the decay (δ).

A machine dynamics model that represents the sample as a simple non-linear spring and piezoelectric load cell as spring, mass, and dashpot was built. Given that the load-displacement response of the sample at the high strain rates is significantly affected by the inertial resistance of the load cell, the spring stiffness of the sample is obtained by fitting the load-displacement response of the sample at a preceding lower strain rate that is already corrected for machine dynamics. The sample stiffness is multiplied by the actuation displacement, corresponding to the current strain rate, to obtain the *mock load*. The *mock load* is then used to obtain the compressive displacement (x) of the load cell using Equation (6). Subsequently, by differentiation with respect to time, the velocity and acceleration of the load cell were also obtained. Using these values in Equation (5), the true load can be calculated. The iterative case study following this protocol starting from 10 s^{-1} till 10^3 s^{-1} is given below as an example:

The load (P)- displacement (h) curve of the 10^1 s^{-1} (slow enough to be not affected by inertial resistance of the load cell) experiment was fit using a quadratic function, $P = C_0h^2 + C_1h$. By

using the parameters C_0 and C_1 along with the compliance-corrected displacement from the 10^2 s^{-1} experiment, a *mock load* was calculated. This *mock load* (F_{mock}) was then used to calculate the internal compressive displacement (x) of the piezoelectric load cell using Equation (6) and by differentiating (x), the acceleration $\ddot{x}$ and velocity $\dot{x}$ were obtained. By substituting these values into Equation (5), the true load response (P_{true}) at 10^2 s^{-1} corrected for inertial resistance of the load cell could be obtained.

Similarly, for correcting the machine dynamics for experiments carried out at 10^3 s^{-1}, the machine dynamics-corrected load-displacement curve of the 10^2 s^{-1} experiment was fitted using the quadratic function to obtain updated C_0 and C_1 parameters. These parameters, along with the compliance-corrected displacement from the 10^3 s^{-1} experiment, were used to generate a *mock load* that provided an accurate measure of the load cell's compressive displacement (x). Using this, the underestimation of load due to the inertial resistance of the load was calculated, and the load-displacement response at 10^3 s^{-1} was corrected for machine dynamics. This same procedure was repeated for each higher strain rate.

The *concise flowchart for machine dynamics correction is as,*

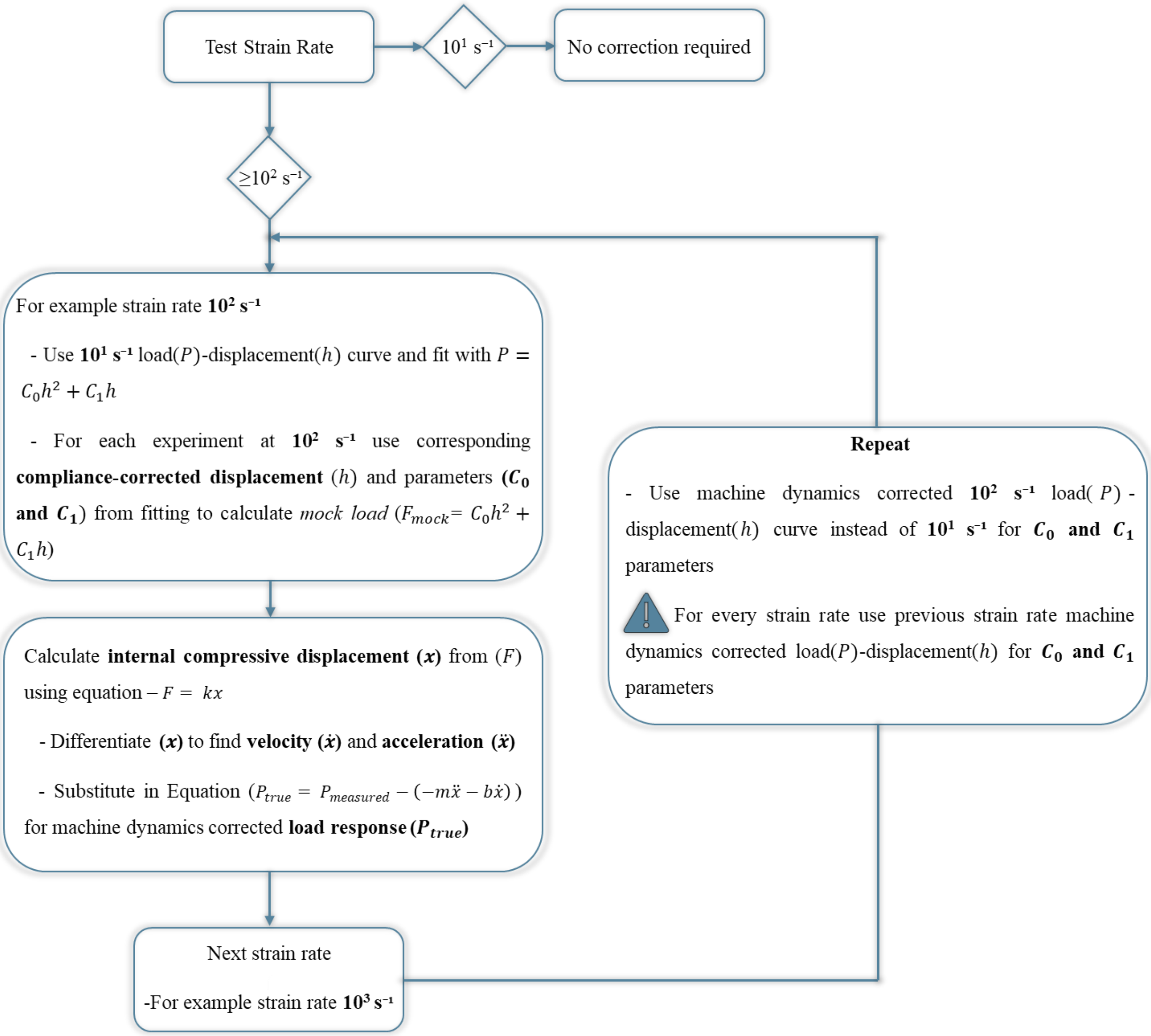

S2.1 Error analysis based on input C_0 and C_1 parameters:

To evaluate the validity of these assumptions, an error analysis was performed on one of the load-displacement curves at the highest strain rate, 10^5 s^{-1} for molybdenum. The first step involves performing a quadratic fit on a machine dynamics-corrected load-displacement curve from a prior strain rate to obtain the C_0 and C_1 parameters as shown in fig. S18A. To examine the influence of these parameters, four different strain rates were selected: the machine dynamics-corrected load-displacement curves of 5×10^4 s^{-1} corrected with 2.5×10^4 s^{-1}, 2.5×10^4 s^{-1} corrected with 10^4 s^{-1}, 10^4 s^{-1} corrected with 3×10^3 s^{-1}, and the load-displacement curve of

10^1 s^{-1}. Additionally, C_0 and C_1 parameters were obtained by quadratic fitting of the uncorrected 10^5 s^{-1} curve. This approach was taken because, in the literature [4], a common simplification for correcting machine dynamics assumes that the measured load ($P_{measured}$) is same as the external force (F) used to calculate the internal displacement (x) of the secondary body. This study was aimed to understand the impact of this assumption. The C_0 and C_1 parameters, along with the compliance-corrected displacement (shown in the inset of fig. S18B), were used in the quadratic function to calculate the *mock load* (F_{mock}), as shown in fig. S18B. This *mock load* (F_{mock}) was used to determine the accurate compressive displacement (x) of the piezoelectric load cell using Equation (6). By differentiating (x) with respect to time, the velocity ($\dot{x}$) and acceleration ($\ddot{x}$) were derived and applying them to Equation (5) the true load response (P_{true}) corrected for machine dynamics could be obtained. Fig. S18C presents the machine dynamics-corrected load-displacement curves for 10^5 s^{-1}, calculated using the different C_0 and C_1 parameters. Although fig. S18C shows minimal differences among the machine dynamics-corrected load-displacement curves, the load difference relative to the curve corrected using C_0 and C_1 parameters from 5×10^4 s^{-1} are compared with the others and plotted in fig. S18D. One can observe that if one uses C_0 and C_1 parameters from 2.5×10^4 s^{-1}, the difference in load at 700 nm is less than ~ 1.3 mN and if it is 10^4 s^{-1}, it is ~ 3.5mN and it becomes significantly higher only if 10^1 s^{-1} is used. Also, if the non-machine dynamic corrected 10^5 s^{-1} is used to obtain the C_0 and C_1 parameters, the difference is in the order of ~ 1.3 mN. For perspective, this load variation leads to a hardness difference of approximately 0.15 GPa when using C_0 and C_1 parameters from 2.5×10^4 s^{-1} and non-machine dynamic corrected 10^5 s^{-1}. The difference increases to about 0.4 GPa and 0.9 GPa when using C_0 and C_1 parameters from 10^4 s^{-1} and 10^1 s^{-1}, respectively. However, it is recommended to use the closest prior strain rate load-displacement response, corrected for machine dynamics, to determine the C_0 and C_1 parameters at high strain rates, thereby minimizing potential errors. An interesting observation is that while the loading portion exhibited changes in load, the peak load value remained unaffected by machine dynamics corrections, as shown in fig. S5B.

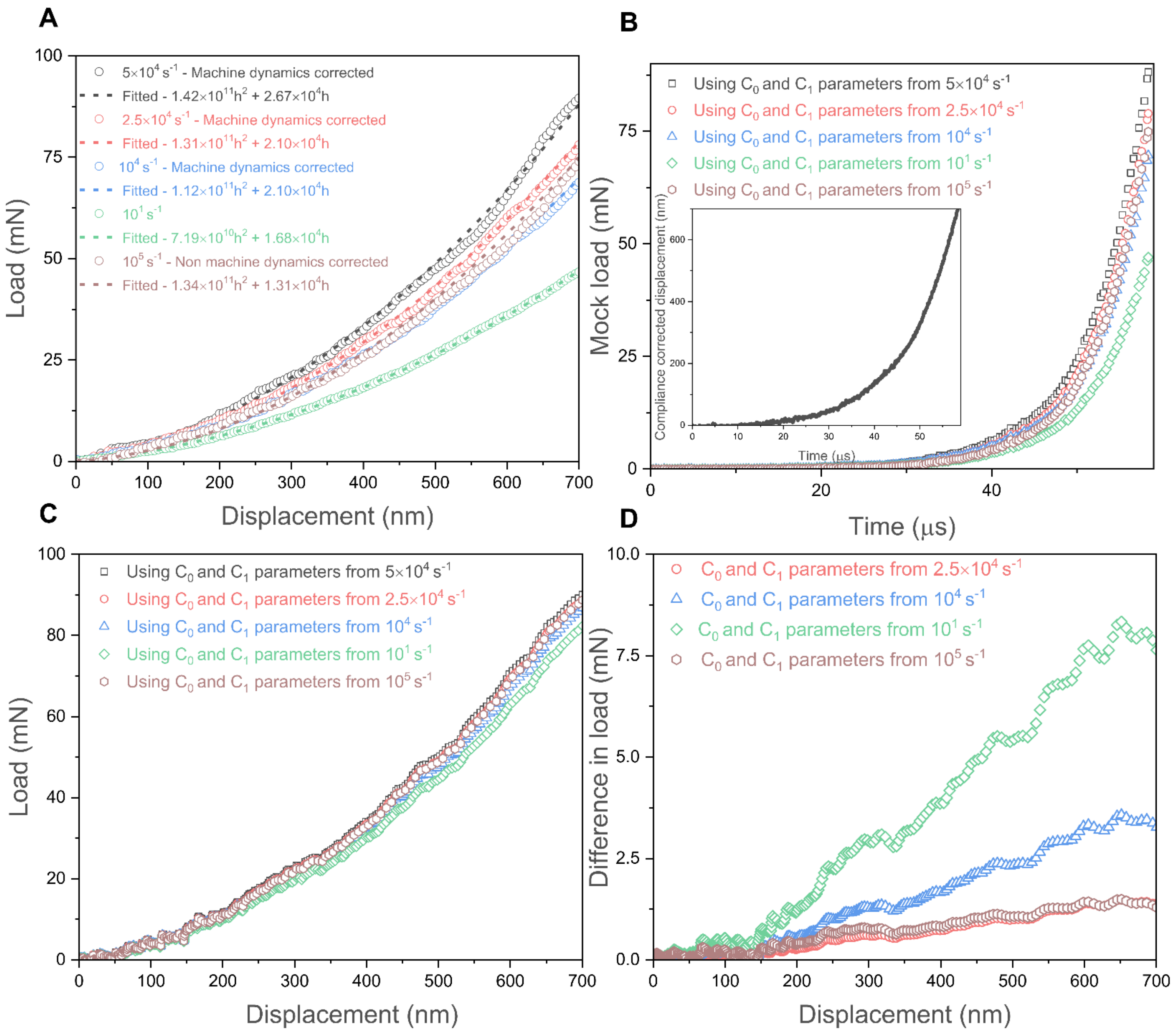

Fig. S18.

(**A**) The various load-displacement curves that were quadratically fitted to obtain the C_0 and C_1 parameters. (**B**) the *mock load* (F_{mock}) calculated using the parameters along with the compliance-corrected displacement (shown in the inset) in the quadratic function. (**C**) the machine dynamics-corrected load-displacement curves for 10^5 s^{-1}, calculated using the C_0 and C_1 parameters obtained from the load-displacement curves in figure (**A**). (**D**) the load difference for all the curves in (c) relative to the curve corrected using C_0 and C_1 parameters from 5×10^4 s^{-1}.

S2.2 Error propagation analysis due to uncertainties in Kelvin–Voigt parameters

In this section, the effect of uncertainties in the damping coefficient (b), elastic spring stiffness (k), and mass (m) on the hardness is assessed. From the estimated Kelvin–Voigt parameters, the damping coefficient (b) = 10.5 $\pm$ 5.5 $\frac{Ns}{m}$, elastic spring stiffness (k) = 3.22E7 N/m, and mass (m) = $0.238\ \pm\ 0.010$ g. The spring stiffness (k) is treated as an absolute value without uncertainty, as it was provided directly by the manufacturer. For the error propagation analysis, the focus is on the highest strain rate (10^5 s^{-1}) load-displacement curve, where the effect of inertia is expected to be most significant. To this end, one representative molybdenum experiment at this strain rate is carefully analyzed under two scenarios: (1) keeping the mass constant while varying the damping coefficient, and (2) keeping the damping coefficient fixed while varying the mass. The resulting changes in the load-displacement response and the corresponding hardness values is then evaluated.

Scenario 1: Keeping the mass constant while varying the damping coefficient

In this case, the mass (m) was fixed at 0.238 g and the damping coefficient was varied from 10.5 $\pm$ 5.5 $\frac{Ns}{m}$. Figure S19A presents the load–displacement curves from a representative molybdenum experiment at a strain rate of 10^5 s^{-1} obtained by varying the damping coefficient, while the corresponding hardness variation is shown in Figure S19B. From the hardness data, the variation in the damping coefficient results in a standard deviation of approximately 0.255 GPa. Although this is smaller than the scatter observed across different experiments at the same strain rate, it is nevertheless accounted for and reflected in the final results reported in the main manuscript (Figure 2B-D).

Scenario 2: Keeping the damping coefficient constant while varying the mass

In this case, damping coefficient was fixed to 10.5 $\frac{Ns}{m}$ and the mass (m) was varied between $0.238 \pm\ 0.010$ g . Figure S19C presents the load–displacement curves from a representative molybdenum experiment at a strain rate of 10^5 s^{-1} obtained by varying the mass, while the corresponding hardness variation is shown in Figure S19D. From the hardness data, the variation in the mass results in an even lower standard deviation when compared to scenario 1 and is approximately 0.089 GPa

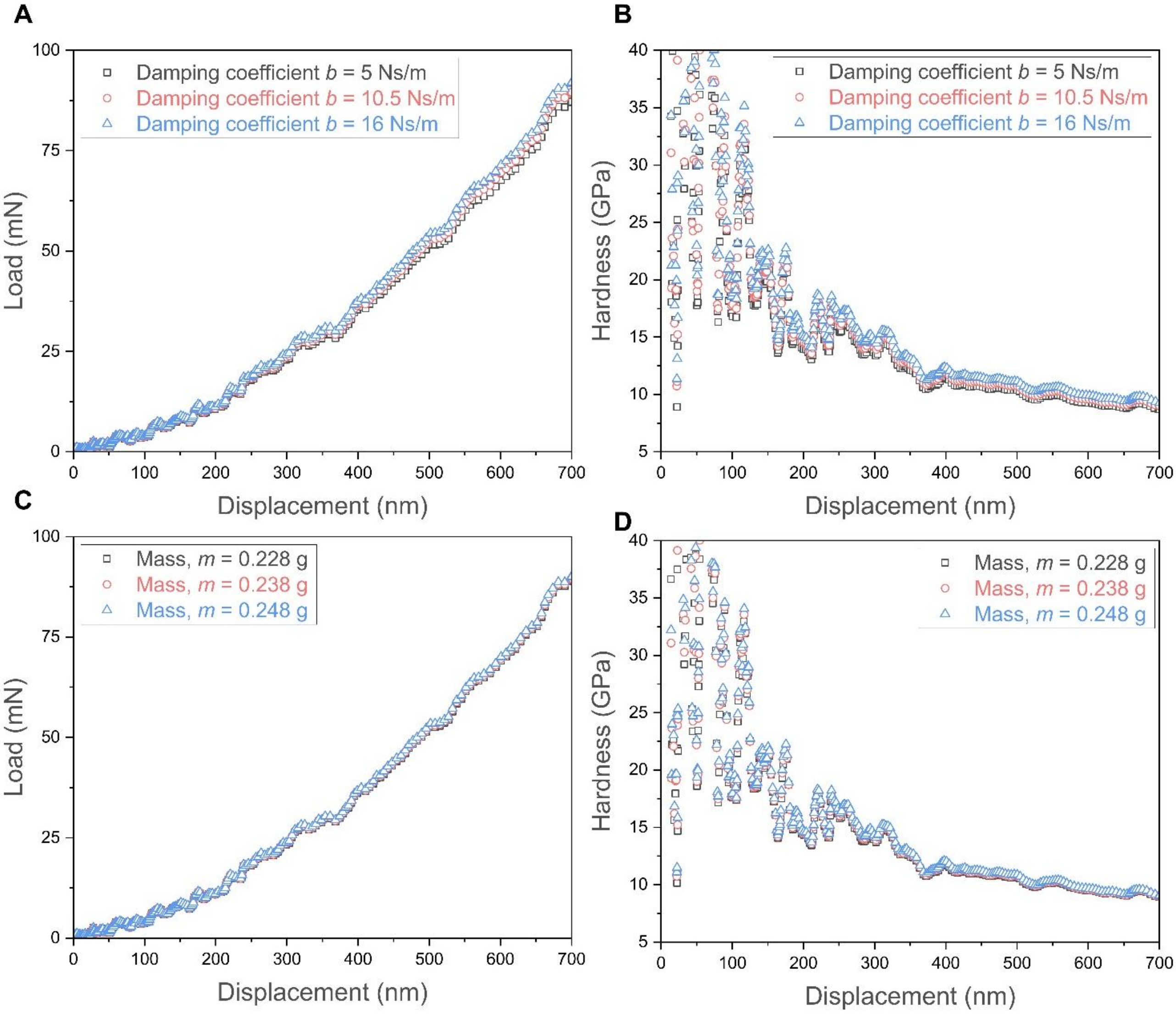

Fig. S19.

Load–displacement curves (**A & C**) and corresponding hardness values (**B & D**) from representative molybdenum experiment at a strain rate of 10^5 s^{-1}, illustrating the effect of uncertainties in the Kelvin–Voigt parameters (damping coefficient (b), and mass (m))

Section S3 - Nanoindentation experiments, micropillar compression testing, quasi-static reloading experiments, hardness measurement, and TEM, along with dislocation density measurement

S3.1 Nanoindentation experiments

All indentation experiments were performed using a Berkovich indenter tip from Synton MDP (Nidau, Switzerland). Tests were conducted across eight different strain rates, ranging from 10^1 s^{-1} to 10^5 s^{-1}. Exponential voltage profiles were used for achieving constant indentation strain rate ($\dot{h}/h$) with the loading and unloading rate remaining the same for each strain rate.

Additionally, there was no holding time at the peak load. For all strain rates, the target indentation depth was consistently set to approximately 1.7 µm. It should be noted that this value represents the displacement, without accounting for system compliance corrections. Given the maximum indentation depth, the loading and unloading rates at each strain rate ($\dot{\varepsilon}$) can be determined using the relation ($\dot{\varepsilon} = \dot{h}/h$, where $\dot{h}$ is loading/unloading rate and h is the input displacement). The calibration factor for the piezo actuator used is 13.5 nm/V. For each strain rate, five independent indentations were performed to ensure statistical relevance. The load–displacement curves were corrected for machine dynamics using the procedure outlined in section S2 (S2 Machine dynamics – Methodology and error analysis) and subsequently averaged using a standard averaging procedure. The curves presented in all figures represent the averaged data, with the corresponding standard deviation included to indicate data scatter. The indenter was brought into contact with the sample using a pre-set load of 500 µN, with careful control to maintain a gap of approximately 10 nm between the tip and the sample before the start of each experiment. System compliance was measured using the multiple indentation method with a constant strain rate ($\dot{h}/h$) of 10^1 s^{-1} prior to conducting the high strain rate indentation experiments on each sample. All indentation experiments were conducted *in situ* within a Zeiss Gemini 500 (SEM) operating at 5 kV. For experiments involving fused silica, the electron beam was switched off during indentation. Indentation at a strain rate of 10^0 s^{-1} on fused silica was performed using the quasi-static setup [7] from Alemnis AG (Switzerland), while indentation at 5×10^{-2} s^{-1} was conducted using the G200 nanoindenter (KLA Corporation, USA) equipped with a standard Berkovich tip and employing the continuous stiffness method (CSM).

S3.2 Micropillar compression testing

Compression tests were performed on lithographically fabricated fused silica micropillars with a diameter of 2.5 µm and height of 5.5 µm, at strain rates ranging from 1 s^{-1} to 1750 s^{-1} and on 5 µm and height of 14.5 µm, at strain rates ranging from 0.3 s^{-1} to 1250 s^{-1}. The maximum compression achieved was 2 µm, at which point most pillars fractured. The load-displacement curves were converted into stress-strain data using the top cross-sectional area and pillar height. Since the majority of deformation occurred in the upper one-third of the pillar, this assumption for the top cross-sectional area in calculating the stress was considered valid. It was ensured that experiments were carried out at constant strain rates. The experiments were conducted *in situ* within a Zeiss Gemini 500 (SEM) operating at 5 kV. Prior to testing, a 30 µm diameter flat

punch tip (from Synton MDP) was aligned with the top of the pillars, after which the electron beam was turned off for the compression experiments.

S3.3 Quasi-static reloading experiments

The reloading experiments were conducted immediately following the high constant strain rate nanoindentation tests. After the initial high strain rate indentations, the indenter was automatically repositioned onto the pre-existing indent with a minimum auto-approach load of 500 μN. Subsequent reloading was performed in a displacement-controlled mode at a constant strain rate using the profile of 10^1 s^{-1}. To ensure consistency, the initial system compliance corrected displacements during the high strain rate indentations were kept uniform across all experiments. The reloading hardness values were extracted from the load-displacement curves using the unloading stiffness based traditional Oliver-Pharr method [8]. It is important to note that, at the highest strain rates, the strain rates were not constant throughout the entire indentation depth (see fig. S1). Nonetheless, reloading experiments were performed on those indents.

S3.4 Hardness measurement

In this study, hardness measurements were conducted using two complementary techniques.

S3.4.1 Projected area method

The projected area method, involved imaging the indents with a VK-X model confocal laser microscope from Keyence Corporation to accurately determine the contact area. The hardness (*H*) was then calculated using the relation:

$$H = \frac{P_{max}}{A_c} \qquad (14)$$

where A_c is the measured contact area, and P_{max} is the maximum load. The imaged indents were analyzed using Gwyddion 2.64 software. A polynomial background correction and baseline correction were done to the confocal images before extracting the contact area of the indents. Any pile-up or sink-in effects that could potentially affect the contact area measurement, and thereby influence the hardness calculation, are accounted for using the calibration method described by K. W. McElhaney *et al.*[9]

The MHP iterative method, is a modification of the traditional Oliver-Pharr equations, as proposed by B. Merle et al [10]. This approach assumes that the reduced elastic modulus E_r remains constant between strain rates. If this assumption becomes invalid, no solution is obtained. Given reliable load-displacement curves and tip area function parameters $\sum_{i=0}^{n} m_i$, hardness as a function of displacement can be determined. The brief description along with equations and MATLAB script for the MHP iterative method are as follows,

S3.4.2 MHP Iterative method

This method is a modification of the traditional Oliver-Pharr equations, as proposed by B. Merle et al[10]. This approach assumes that the reduced elastic modulus (E_r) remains constant between strain rates. From standard Oliver–Pharr equations [11] the contact stiffness (S) and contact depth (h_c) is given by the following equation,

$$S = \frac{2E_r\beta\sqrt{A_c}}{\sqrt{\pi}} \qquad (15)$$

$$h_c = h - \varepsilon\frac{P}{S} \qquad (16)$$

Where, β and ε are constants dependent on the indenter geometry, with values close to 1.0 and 0.75, respectively. And the contact area (A_c) is given by,

$$A_c = \sum_{i=0}^{n} m_i h_c^{2^{1-i}} \ (17)$$

In this study, three contact area parameters (m_0 = 25.03886, m_1 = -5.53811E-7, and m_2 = 3.25942E-10) were utilized to model the tip shape accurately. These parameters were derived using continuous stiffness measurement (CSM) on fused silica at a strain rate of 10^{-2} s^{-1} with an oscillation frequency of 10 Hz and an amplitude of 20 nm, performed using a quasi-static testing setup, detailed elsewhere [7].

Using equations (15-17), the equation was re-written to the following form to eliminate the contact stiffness (S),

$$\left\{\left(\sum_{i=0}^{n} m_i h_c^{2^{1-i}}\right) \times (h_c - h)^2\right\} - \frac{\pi\varepsilon^2 P^2}{4\beta^2 {E_r}^2} = 0 \qquad (18)$$

Using an iterative technique, a solution for h_c less than the indentation depth (h) was identified. From the h_c, A_c was calculated using equation (17). Then using the following equation hardness (H) was calculated,

$$H = \frac{P}{A_c} \qquad (19)$$

where A_c is the measured contact area, and P is the applied load. Using this method, hardness could be calculated continuously over the entire displacement.

The MATLAB script for the iterative method is as follows,

```
% The input file should be in .CSV format, with the 1st row as time(s), 2nd row
% as displacement(m) and 3rd row as load(N)
```

```
clc;
clear all;
format shortE;
M = readtable('inputfilename.csv', 'DecimalSeparator',',');
M = M{:,:};
Ti = M(:,1);
Disp = M(:,2);
Load = M(:,3);
Di = smoothdata(Disp,"sgolay",50);
Lo = smoothdata(Load,"sgolay",50);
Er =; % enter reduced elastic modulus here
m0 =;% enter contact area parameters
m1=;% enter contact area parameters
m2=;% enter contact area parameters
L=length(Di);
Z=0;
j1=0;
for i1 = 1:1:L
    Z = Z+1;
    d = Di(i1,1);
    l = Lo(i1,1);
    in = d/100000;
    j=0;
    for x = 0:in:d
    j=j+1;
    h(j,1)= x;
    y(j,1)= (((m0*(x^2))+ (x*m1)+ ((x^0.5)*m2))*((d-x)^2)) -
((3.14*0.5625*(l^2))/(4.276624*(Er^2)));
    end
    zci = @(v) find(diff(sign(v))); % Returns Approximate Zero-Crossing Indices Of
Argument Vector
    zx = zci(y);
    M = max (zx);
    Sol = h(M,1);
    hc = polyval(Sol,1);
    Ac = ((m0*(hc^2))+ (hc*m1)+ ((hc^0.5)*m2));
    Hard = l/Ac;
    j1=j1+1;
    H(j1,1) = d;
    H(j1,2) = Hard;
    H(j1,3) = hc;
    H(j1,4) = Ac;
end
plot(H(:,1),H(:,2))
head={'Displacement [m]' 'Hardness [GPa]' 'hc [m]' 'Ac [m2]' };
F=[head;num2cell(H)];
writecell(F,'filenametosave.csv','Delimiter','\t')
```

S3.5 TEM and dislocation density calculations

Lamellae for TEM analysis were prepared using a Thermofischer Scientific Scios 2 focused ion beam-scanning electron microscope (FIB-SEM) equipped with a Ga-ion beam. Lamellae were lifted out from the central regions of the indents (along the median axis of the indent triangle). To ensure consistent cross-sectional orientations, all lamella were prepared in the same direction relative to the indents. The lift-out process was performed at an accelerating

voltage of 30 kV, followed by final cleaning at 5 kV and 48 pA. Annular bright field scanning transmission electron microscopy (ABF-STEM), selected area electron diffraction (SAED), and convergent beam electron diffraction (CBED) was performed on a Thermo Scientific Titan Themis at 300 keV with a convergence angle of 4 mrad. Correlative images were simulated using the program Dr. Probe [12] to find the thickness of the lamella.

Dislocation density measurements were performed using ABF-STEM images. The detailed procedure for extracting dislocation density from a lamella indented at 10^1 s^{-1} is provided, and a similar methodology was used for lamellae indented at 3×10^3 s^{-1} and 5×10^4 s^{-1}. Due to the extremely high dislocation density, as qualitatively evident from the ABF-STEM images in Fig. 4 of the main paper, the traditional line intercept method was not employed. Instead, an alternate method was adopted that calculates the area fraction of pixels covered by dislocations in a sample of given thickness 't'. This approach is detailed by J. Gallet *et al.*[13]. According, to this technique the dislocation density is calculated using the following formulation,

$$\rho = \frac{N^{dislo}}{N^{tot} \times t \times E^{app}} \qquad (20)$$

where, N^{dislo} is the total number of dislocation pixels, N^{tot} is the total number of pixels in the analysed image and E^{app} refers to the apparent dislocation width (in nm) measured from the image at the same magnification used for dislocation density measurements.

Fig. S20A shows the ABF-STEM image of the 10^1 s^{-1} indent, focusing on a region approximately 1.5 μm below the indent tip. For dislocation density measurement, a 1.5 × 1.5 μm^2 area within this region was selected, as shown in fig. S20B. The region 1.5 μm below the indent was chosen because, at higher strain rates, the dislocation density is so high that quantifying it reliably becomes challenging. To maintain consistency across all analyzed indents, the same depth below the indent was selected. After which a threshold was applied in the grayscale value between 0-100 and the ratio of $\frac{N^{dislo}}{N^{tot}}$ was calculated as shown in fig. S20C. The grayscale value was kept constant across all analysed strain rates. Similarly, E^{app} was determined by averaging the apparent dislocation width across several dislocations at the same magnification used for dislocation density measurements, as shown in fig. S20D. By substituting all the measured values, along with the lamella thickness determined from the CBED analysis, into equation (20), the dislocation density was calculated.

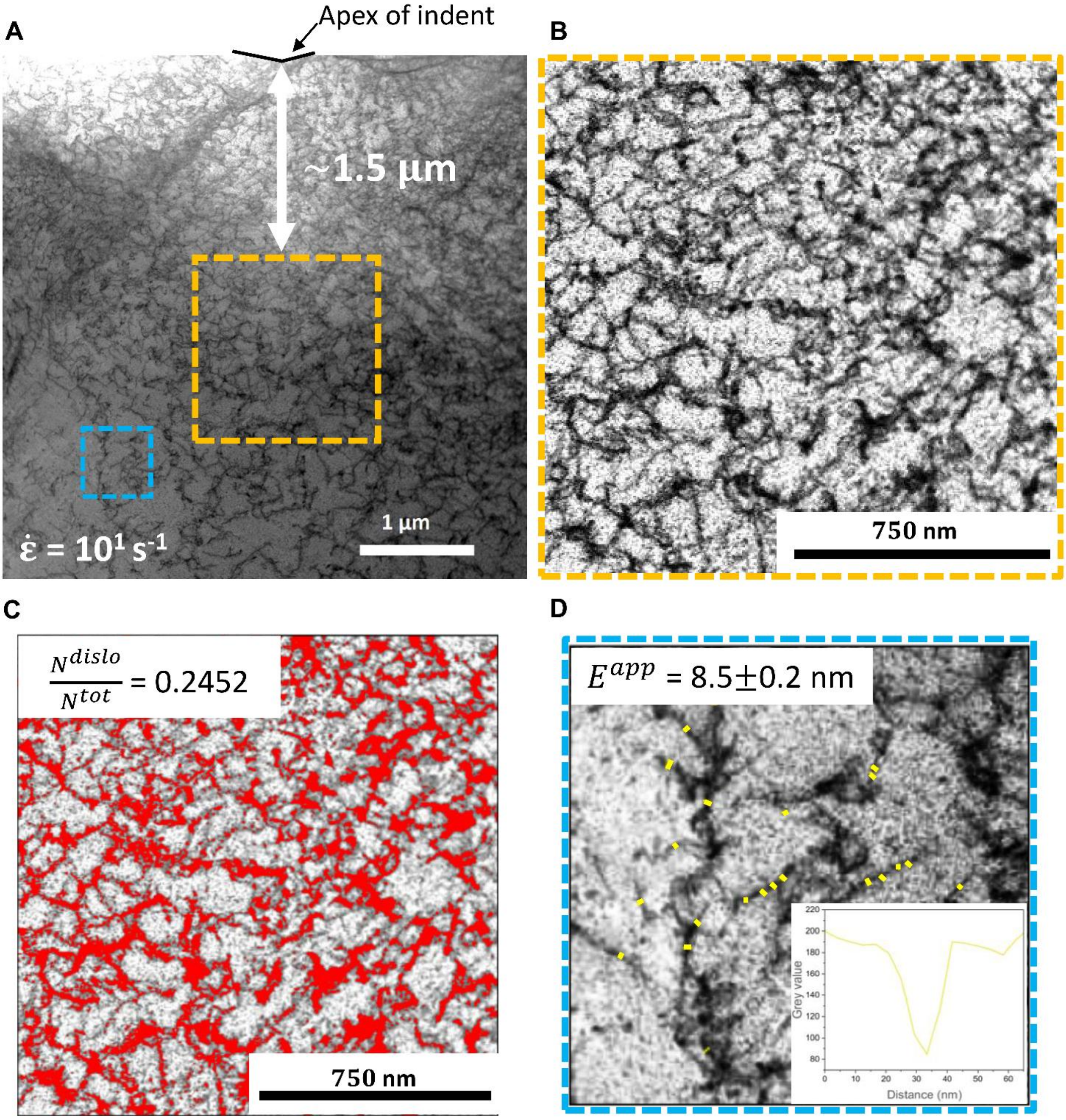

Fig. S20.

(**A**) the ABF-STEM image of the $10^1\ s^{-1}$ (**B**) a region of 1.5 × 1.5 μm^2 area at a distance of 1.5 µm from the indent tip (marked with black lines) (**C**) the same region as in (b) with a grayscale threshold applied (0–100) to determine the $\frac{N^{dislo}}{N^{tot}}$ fraction and (**D**) a selected area from (**A**) used to measure the apparent dislocation width (E^{app}), where the yellow lines indicate the dislocations on which the width measurements were made using a line profile as shown in inset.

To provide further confidence, a detailed dislocation density analysis at various depths, starting from 1 μm, has now been performed using the methodology outlined just above. At each depth increment of 1 μm, an area of 1 × 1 μm² was analyzed, up to a maximum depth of 3 μm. For reference, the maximum indentation depth is approximately 1.25 μm. Furthermore, fig. S21D presents the quantified variation of dislocation density with depth for different strain rates.

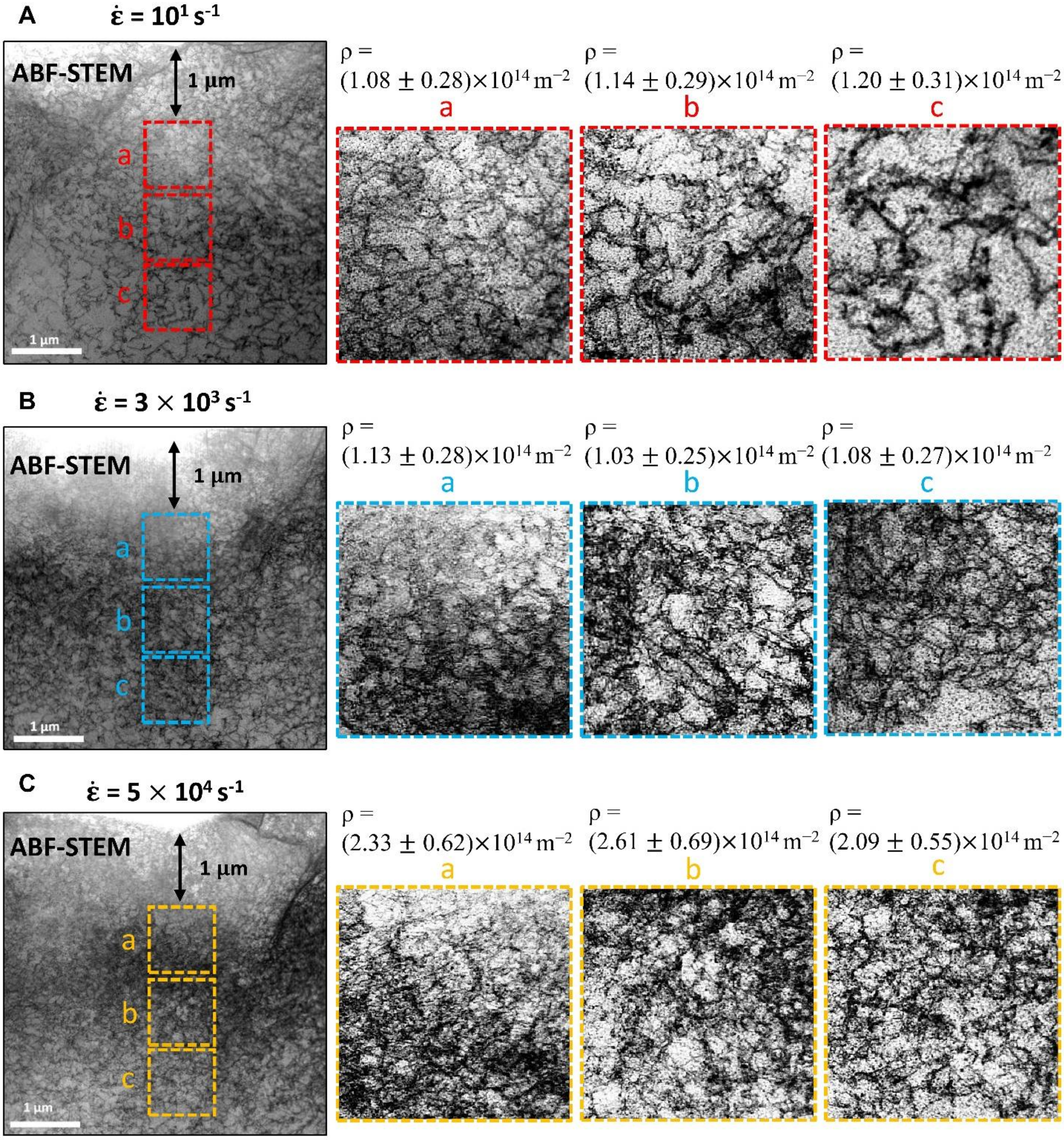

D

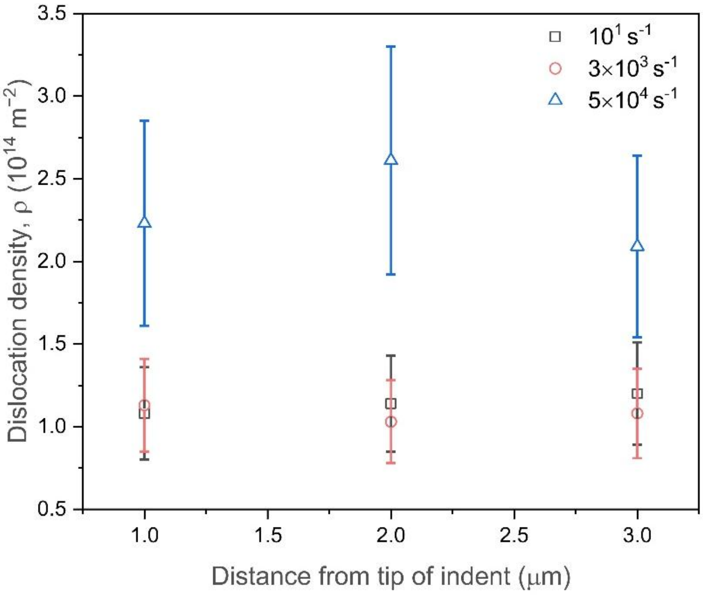

Fig. S21.

Dislocation density analysis from 1 to 3 μm indentation depth (at 1 μm intervals over 1 × 1 μm^2 areas) for indents carried out at strain rates (A) 10^1 s^{-1}, (B) 3 × 10^3 s^{-1} and (C) 5 × 10^4 s^{-1}. (D) shows the variation of dislocation density with depth for different strain rates.

References

[1] M. F. Harold, J.F., Ashby, *Deformation of Mechanism Maps, The Plasticity and Creep of Metals and Ceramics.*, Pergamon Press, **1982**.

[2] M. A. Meyers, K. K. Chawla, *Mechanical Behavior of Materials*, Cambridge University Press, **2019**.

[3] I. Dowding, C. A. Schuh, *Nature* **2024**, *630*, 91.

[4] B. L. Hackett, P. Sudharshan Phani, C. C. Walker, W. C. Oliver, G. M. Pharr, *Jom* **2024**, DOI 10.1007/s11837-023-06338-9.

[5] J. L. Humar, *Dynamics of Structures*, CRC Press, **2002**.

[6] R. Gaillac, P. Pullumbi, F. Coudert, **2016**, DOI 10.1088/0953-8984/28/27/275201.

[7] G. Guillonneau, M. Mieszala, J. Wehrs, J. Schwiedrzik, S. Grop, D. Frey, L. Philippe, J. M. Breguet, J. Michler, J. M. Wheeler, *Mater. Des.* **2018**, *148*, 39.

[8] W. C. Oliver, G. M. Pharr, *J. Mater. Res.* **1992**, *7*, 1564.

[9] K. W. McElhaney, J. J. Vlassak, W. D. Nix, *J. Mater. Res.* **1998**, *13*, 1300.

[10] B. Merle, W. H. Higgins, G. M. Pharr, *J. Mater. Res.* **2020**, DOI

10.1557/jmr.2019.408.

[11] W. C. Oliver, G. M. Pharr, *J. Mater. Res.* **2004**, *19*, 3.

[12] J. Barthel, *Ultramicroscopy* **2018**, *193*, 1.

[13] E. Bouzy, T. Chaise, S. Cazottes, *Mater. Charact.* **2023**, *199*.
